# Novel Phenethylamine-Based Aroyl Thiourea Derivatives: Design, Synthesis, and Multibiological Evaluation

**DOI:** 10.1021/acsomega.5c11616

**Published:** 2026-03-17

**Authors:** Bunyamin Ozgeris, Elif Aksakal, Arzu Gormez

**Affiliations:** † Department of Basic Sciences, Faculty of Science, 226840 Erzurum Technical University, Erzurum 25050, Turkey; ‡ Department of Molecular Biology and Genetics, Faculty of Science, Erzurum Technical University, Erzurum 25050, Turkey; § Department of Biology, Faculty of Science, Dokuz Eylul University, Izmir 35390, Turkey

## Abstract

Drug discovery remains a major global challenge despite significant advances in science and technology that have accelerated drug development efforts. To address this need, numerous studies have focused on designing novel drug molecules with improved efficacy, low toxicity, and cost-effectiveness. Thioureas and their derivatives play a crucial role in medicinal chemistry due to their diverse biological properties, including anticancer, antioxidant, antimicrobial, and anti-inflammatory effects. In this study, seven aroyl thiourea compounds based on substituted phenethylamine were synthesized. Their anticancer, antioxidant, and antibacterial activities were evaluated along with their physicochemical and drug-like properties through *in silico* analysis using the SwissADME software. Among the synthesized compounds, compound **14**, a novel aroyl thiourea derivative, exhibited higher antiproliferative activity than the positive control methotrexate (MTX) against A549 cells, whereas the compounds generally showed moderate antiproliferative effects in HeLa cells. None of the compounds showed cytotoxic effects on the healthy HDF-1 cell lines. Antioxidant activities were determined using CUPRAC and DPPH assays, revealing that compound **10** had the most notable antioxidant capacity in both tests. Additionally, compounds **12** and **15** demonstrated notable antibacterial activity against the bacterial isolates. Overall, *in silico* results indicated that all synthesized aroyl thiourea compounds possess favorable physicochemical characteristics, and it has been determined that they may have potential as medicines.

## Graphical abstract

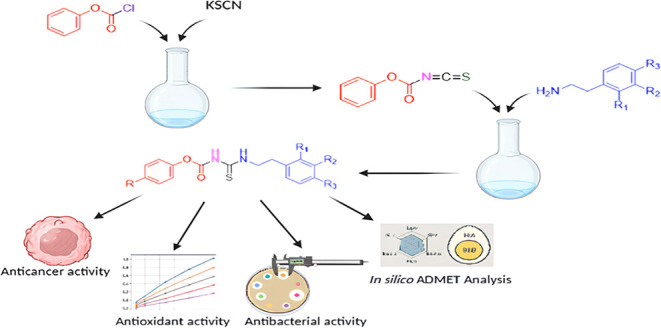

## 1 Introduction

Thiocarbamides, a class of organic compounds with the general formula N–(C=S)–N, differ from ureas by containing a sulfur atom instead of an oxygen atom in their structure.^1^ A thiourea molecule possesses several binding sites, including hydrogen bonding (NH), correlative (S), and secondary binding sites.^2^ Since sulfur is a weak hydrogen bond acceptor, it can hinder hydrogen bond formation; however, the bidentate binding nature of thiourea protons enables simultaneous coordination at two distinct particular.^3^ Thiourea derivatives have found widespread applications in various areas of chemistry and serve as valuable precursors, particularly in the synthesis of heterocyclic compounds.^4^ Aroyl thioureas containing both carbonyl and thiocarbonyl groups can coordinate to transition metals via oxygen and sulfur atoms.^5^ Numerous thiourea derivatives reported in the literature exhibit diverse biological activities, including antitumor, anticancer,^6^ antimicrobial,^7^ antiviral,^8^ anti-inflammatory,^9^ antihypertensive,^10^ antiparasitic,^11^ insecticidal,^12^ herbicidal,^13^ pesticidal, fungicidal,^14^ antioxidant, antidiabetic, and urease inhibitory effects^15^ (Figure 1). These broad-spectrum pharmacological properties render thiourea derivatives highly promising and valuable candidates for drug design and development.

**Figure 1:**
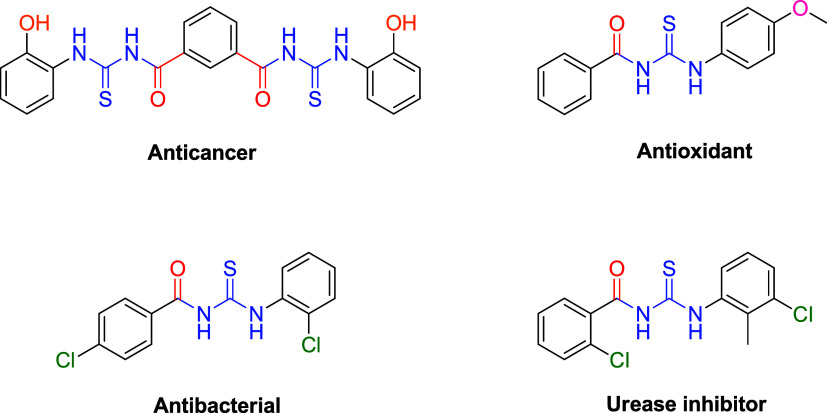
Structures of some biologically active aroyl thiourea derivatives.

Phenethylamine, a trace amine and a natural monoamine alkaloid functioning as a neurotransmitter in the human central nervous system, plays a critical role in neuromodulation.^16^ Structurally, it consists of two saturated carbon atoms, an amino group, and a benzene ring.^17^ Understanding the biological roles of these amines is important in neurological and psychiatric disorders such as epilepsy, Parkinson’s disease, attention deficit hyperactivity disorder, depression, schizophrenia, and Reye’s syndrome. This understanding can be better elucidated by determining their levels in the brain.^18^ Phenethylamines are structural backbones of nearly 200 neurologically active molecules, including neurotransmitters such as serotonin, dopamine, adrenaline, as well as various psychoactive agents.^19^ These compounds have been reported to exhibit significant anticancer,^20^ antioxidant,^21^ and antimicrobial^22^ activities.

Considering the synthetic and biological importance of thioureas and phenethylamine scaffolds, phenethylamine-based aroyl thiourea derivatives were designed and synthesized. Their anticancer/cytotoxic, antioxidant, and antibacterial activities were evaluated, and their physicochemical properties and drug-likeness were predicted using the SwissADME software tool.

## 2 Materials and Methods

### 2.1 Materials

Phenyl chloroformate (Acros), phenethylamine (Acros Organics), 2-methoxyphenethylamine (Thermo scientific), 3-methoxyphenethylamine (Alfa Aesar), 4-methoxyphenethylamine (J&K Scientific), 4-methylphenethylamine (Acros Organics), 4-fluorophenethylamine (J&K Scientific), 3,4-dimethoxyphenethylamine (J&K Scientific), methylene chloride (Carlo Erba), ethyl acetate (Carlo Erba), *n*-hexane (VWR Chemicals BDH), acetone (Carlo Erba), and potassium thiocyanate (Merck) were obtained and used for the synthesis of aroyl thiourea derivatives. For biological activity studies, Dulbecco’s modified Eagle’s medium (DMEM) (Biowest), RPMI-1640 (Biowest), fetal bovine serum (FBS) (Gibco), l-Glutamine (WISENT, Inc.), penicillin-streptomycin (Gibco), phosphate-buffered saline (PBS) (Gibco), Trypsin/EDTA (Gibco), WST-8 (Ecotech, Cat No: CVDK-8), Luria–Bertani Agar (LBA) (Miller MERCK), Potato Dextrose Agar (PDA) (Oxoid), Mueller-Hinton Agar (MHA) (Oxoid), Mueller-Hinton Broth (MHB) (Biolife), ampicillin (Sigma-Aldrich), and dimethyl sulfoxide (DMSO) (Merck) were used. In this study, ^1^H and ^13^C nuclear magnetic resonance (NMR) spectra were recorded in deuterated chloroform (CDCl_3_, Sigma-Aldrich) solvent using 60 MHz (Oxford Instruments Pulsar) and 400 MHz (Varian Mercury) spectrometers. Chemical shifts (δ) are reported in parts per million (ppm). Structural characterization was also confirmed by Fourier transform infrared (FT-IR) spectroscopy (IRTracer-100), and melting points were determined by using a capillary melting point apparatus (Electrothermal IA9100). Spectrophotometric measurements for biological assays were performed using a microplate reader (Epoch spectrophotometer).

### 2.2 Synthesis

Phenyl chloroformate (**1**) (3.18 mmol) was dissolved in 30 mL of acetone and reacted with potassium thiocyanate (1.0 mmol) dissolved in 30 mL of acetone at 75 °C for 1 h to afford the corresponding carbonyl isothiocyanate (**2**). To the resulting carbonyl isothiocyanate (**2**) (1.0 mmol), substituted phenethylamines (**3**–**9**) (1.0 mmol, each dissolved in 30 mL of acetone) were added, and the reaction mixture was stirred at 70 °C for an additional 2 h. After the completion of the reaction, potassium chloride (KCl) precipitated as a byproduct. The reaction mixture was filtered through filter paper to remove the solid residue, and the filtrate was evaporated under reduced pressure by using a rotary evaporator. The resulting aroyl thiourea derivatives **10**–**16** were dissolved in methylene chloride, purified by column chromatography, and crystallized from methylene chloride/hexane to afford the pure compounds in yields ranging from 27 to 99% ^23,24^ (Scheme 1). The spectral and analytical data of the synthesized compounds are provided below.

**Scheme 1:**
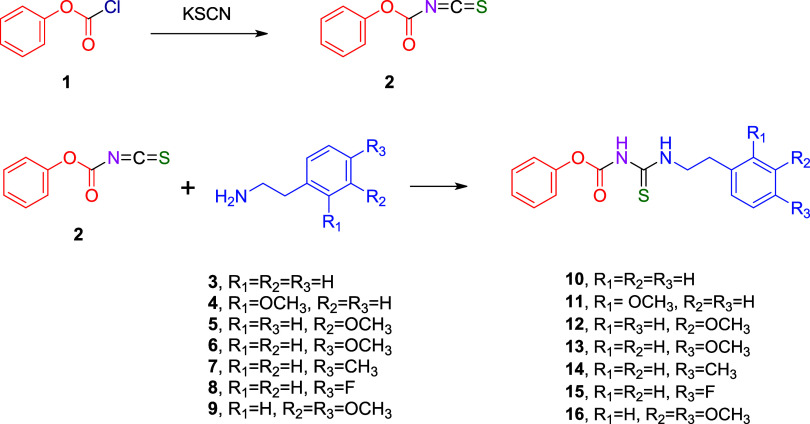
Synthesis Design of Aroyl Thioureas

#### 2.2.1 *N*-Phenethyl-*N*′-(phenoxycarbonyl) Thiocarbamide (**10**)

White solid. Yield: (0.919 g, 98%); Melting point: 111–113 °C. ^**1**^ **H NMR (400 MHz**, **CDCl**_**3**_ **)** δ 9.58 (s, 1H, NH), 8.42 (s, 1H, NH), 7.41 (t, *J* = 7.9 Hz, 2H, ArH), 7.33–7.20 (m, 6H, ArH), 7.12 (d, *J* = 7.7 Hz, 2H, ArH), 3.93 (dd, *J* = 12.7, 7.2 Hz, 2H, CH_2_), 2.98 (t, *J* = 7.3 Hz, 2H, CH_2_). ^**13**^ **C NMR (100 MHz**, **CDCl**_**3**_ **)** δ 179.0 (CS) 151.2 (CO), 149.7 (C), 138.2 (C), 129.9 (2CH), 129.0 (2CH), 128.9 (2CH), 127.0 (CH), 126.9 (CH), 121.5 (2CH), 47.3 (CH_2_), 34.6 (CH_2_). **FTIR (cm** ^**–1**^ **)**: 3275 (N–H), 1716 (C=O), 1521,1489 (N–H, C–H), 1454, 1402 (C=C), 1332, 1220 (C=S), 1149,1001 (C–N, C–O), 910, 844, 794, 740 (Ar–CH), 684, 653 (C–S).

#### 2.2.2 *N*-(2-Methoxyphenethyl)-*N*′-(phenoxycarbonyl) Thiocarbamide (**11**)

White solid. Yield (0.342 g, 33%); Melting point: 118–120 °C. ^**1**^ **H NMR (400 MHz**, **CDCl**_**3**_ **)** δ 9.56 (s, 1H, NH), 8.44 (s, 1H, NH), 7.47–7.34 (m, 2H, ArH), 7.33–7.22 (m, 1H, ArH), 7.17–7.06 (m, 4H, ArH), 6.88–6.77 (m, 2H, ArH), 3.94–3.84 (m, 2H, CH_2_), 3.79 (s, 3H, OCH_3_), 2.98–2.83 (m, 2H, CH_2_). ^**13**^ **C NMR (100 MHz**, **CDCl**_**3**_ **)** δ 179.0 (CS), 158.6 (CO), 151.2 (C), 149.7 (C), 130.2 (C), 129.9 (2CH + CH, overlapped), 126.9 (CH), 121.5 (2CH + CH, overlapped), 114.4 (CH + CH, overlapped), 55.5 (OCH_3_), 47.5 (CH_2_), 33.7 (CH_2_). **FTIR (cm** ^**–1**^ **)**: 3242 (N–H), 2933 (C–H), 1716 (C=O), 1610 (C–N, N–H), 1521, 1510, 1489 (C–N, N–H), 1328, 1298, 1220 (C=S), 1151, 1111, 1035, 1001 (C–N, C–O), 912, 817, 756, 719 (Ar–CH), 650 (C–S).

#### 2.2.3 *N*-(3-Methoxyphenethyl)-*N*′-(phenoxycarbonyl) Thiocarbamide (**12**)

Yellow crystalline. Yield (1.04 g, 99%); Melting point: 137–139 °C. ^**1**^ **H NMR (400 MHz**, **CDCl**_**3**_ **)** δ 9.58 (s, 1H, NH), 8.35 (s, 1H, NH), 7.41 (t, *J* = 7.8 Hz, 2H, ArH), 7.28 (dd, *J* = 12.5, 5.4 Hz, 1H, ArH), 7.21 (d, *J* = 7.5 Hz, 1H, ArH), 7.14–7.10 (m, 2H, ArH), 6.84–6.75 (m, 3H, ArH), 3.96–3.88 (m, 2H, CH_2_), 3.78 (s, 3H, OCH_3_), 2.95 (t, *J* = 7.2 Hz, 2H, CH_2_). ^**13**^ **C NMR (100 MHz**, **CDCl**_**3**_ **)** δ 179.0 (CS), 160.1 (CO), 151.2 (C), 149.7 (C), 139.8 (C), 130.0 (CH), 129.9 (2CH), 126.9 (CH), 121.5 (2CH), 121.2 (CH), 114.4 (CH), 112.6 (CH), 55.4 (OCH_3_), 47.2 (CH_2_), 34.6 (CH_2_). **FTIR (cm** ^**–1**^ **)**: 3267, 3170, 3032 (N–H), 1724 (C=O), 1593, 1533 (N–H, C–N), 1479, 1452 (C=C), 1398 (C–N), 1261, 1215, 1153 (C=S), 1031, 991, 916, 854 (C–H), 790, 732, 688 (Ar–H), 599 (C–S).

#### 2.2.4 *N*-(4-Methoxyphenethyl)-*N*′-(phenoxycarbonyl) Thiocarbamide (**13**)

White solid. Yield (0.450 g, 43%); Melting point: 151–153 °C. ^**1**^ **H NMR (400 MHz**, **CDCl**_**3**_ **)** δ 9.57 (s, 1H, NH), 8.55 (s, 1H, NH), 7.43–7.37 (m, 2H, ArH), 7.32–7.25 (m, 1H, ArH), 7.17–7.09 (m, 4H, ArH), 6.87–6.81 (m, 2H, ArH), 3.89 (td, *J* = 7.2, 5.5 Hz, 2H, CH_2_), 3.78 (s, 3H, OCH_3_), 2.99–2.82 (m, 2H, CH_2_). ^**13**^ **C NMR (100 MHz**, **CDCl**_**3**_ **)** δ 179.0 (CS), 158.6 (CO), 151.3 (C), 149.7 (C), 130.2 (C), 129.9 (2 × 2CH, overlapped), 126.9 (CH), 121.5 (2CH), 114.4 (2CH), 55.5 (OCH_3_), 47.5 (CH_2_), 33.7 (CH_2_). **FTIR (cm** ^**–1**^ **)**: 3236 (N–H), 2933 (C–H), 1716 (C=O), 1610, 1541 (N–H, C–N), 1481, 1456 (C=C), 1329, 1298, 1222 (C–N, C=S), 1151 (C–N), 1109–817 (Ar–CH), 756, 719, 669, 650 (Ar–H), 555 (C–S).

#### 2.2.5 *N*-(4-Methylphenethyl)-*N*′-(phenoxycarbonyl) Thiocarbamide (**14**)

White solid. Yield (0.272 g, 27%); Melting point: 143–145 °C. ^**1**^ **H NMR (400 MHz**, **CDCl**_**3**_ **)** δ 9.59 (s, 1H, NH), 8.51 (s, 1H, NH), 7.16–7.07 (m, 5H, ArH), 7.07–6.99 (m, 2H, ArH), 6.95–6.86 (m, 2H, ArH), 3.95–3.84 (m, 2H, CH2), 2.93 (t, *J* = 7.2 Hz, 2H, CH2), 2.31 (s, 3H, CH3). ^**13**^ **C NMR (100 MHz**, **CDCl**_**3**_ **)** δ 179.0 (CS), 158.0 (CO), 151.6 (C), 136.5 (C), 135.1 (C), 129.6 (2CH), 128.8 (2CH), 126.9 (CH), 122.4 (2CH), 114.8 (2CH), 47.4 (CH_2_), 34.1 (CH_2_), 21.3 (CH_3_). **FTIR (cm** ^**–1**^ **)**: 3255 (N–H), 2924 (C–H), 1712 (C=O), 1539,1496, 1454 (N–H, C–N), 1454, 1398, 1328 (Ar C=C, C–N), 1219 (C–N), 1031–790 (Ar–CH), 547 (C–S).

#### 2.2.6 *N*-(4-Fluorophenethyl)-*N*′-(phenoxycarbonyl) Thiocarbamide (**15**)

White powdery. Yield (1.55 g, 77%); Melting point: 115–117 °C. ^**1**^ **H NMR (400 MHz**, **CDCl**_**3**_ **)** δ 9.60 (s, 1H, NH), 8.68 (s, 1H, NH), 7.41 (dd, *J* = 10.8, 5.0 Hz, 2H, ArH), 7.28 (dd, *J* = 12.6, 5.1 Hz, 1H, ArH), 7.17 (ddd, *J* = 24.0, 13.7, 4.8 Hz, 4H, ArH), 7.03–6.94 (m, 2H, ArH), 3.90 (dd, *J* = 12.7, 7.2 Hz, 2H, CH2), 2.95 (t, *J* = 7.3 Hz, 2H, CH2). ^**13**^ **C NMR (100 MHz**, **CDCl**_**3**_ **)** δ 179.2 (CS), 163.2 (CO), 151.4 (C), 149.7 (C), 133.9 (C), 130.4 (2CH), 129.9 (2CH), 126.9 (CH), 121.5 (2CH), 115.9 (2CH), 47.2 (CH_2_), 33.7 (CH_2_). **FTIR (cm** ^**–1**^ **)**: 3269 (N–H), 1716 (C=O), 1556, 1506 (C=S, C–N), 1328, 1217, 1151 (C–N), 848–684 (Ar–CH), 655, 551 (C–S).

#### 2.2.7 *N*-(3,4-Dimethoxyphenethyl)-*N*′-(phenoxycarbonyl) Thiocarbamide (**16**)

White solid. Yield (0.628 g, 28%); Melting point: 97–99 °C. ^**1**^ **H NMR (400 MHz**, **CDCl**_**3**_ **)** δ 9.58 (s, 1H, ArH), 8.41 (s, 1H, ArH), 7.44–7.35 (m, 2H, ArH), 7.31–7.23 (m, 1H, ArH), 7.11 (dd, *J* = 5.7, 3.8 Hz, 2H, ArH), 6.82–6.71 (m, 3H, ArH), 3.90 (dd, *J* = 12.4, 7.1 Hz, 2H, CH_2_), 3.843 (s, 3H, OCH_3_), 3.840 (s, 3H, OCH_3_), 2.91 (t, *J* = 7.1 Hz, 2H, CH_2_). ^**13**^ **C NMR (100 MHz**, **CDCl**_**3**_ **)** δ 178.9 (CS), 151.2 (CO), 149.7 (C), 149.2 (C), 148.0 (C), 130.7 (C), 129.9 (2CH), 126.9 (CH), 121.5 (2CH), 120.8 (CH), 112.1 (CH), 111.6 (CH), 56.1 (OCH_3_), 56.0 (OCH_3_), 47.5 (CH_2_), 34.2 (CH_2_). **FTIR (cm** ^**–1**^ **)**: 3280, 3159, 3032 (N–H), 1724 (C=O), 1548, 1516 (N–H, C–N), 1456, 1319 (C–N, C=S–N), 1261, 1217, 1178, 1134 (C=S), 1024, 999, 889 (C–H), 798, 761, 744, 686 (Ar–CH), 603 (C–S).

### 2.3 Toxicity Research

#### 2.3.1 Cell Culture

Cancer cell lines A549 (cultured in RPMI-1640 medium) and HeLa (cultured in DMEM) along with healthy cell line HDF-1 (cultured in DMEM), were maintained in a medium containing 10% FBS, 1% l-glutamine, and 1% penicillin/streptomycin. All cells were incubated at 37 °C in a 5% CO_2_ incubator for proliferation. All cell lines were obtained from the High Technology Application and Research Center (YUTAM) at Erzurum Technical University and were validated and routinely tested for mycoplasma contamination.

#### 2.3.2 WST-8 Cell Viability Assay

The water-soluble tetrazolium salt-8 (WST-8) assay is a colorimetric cytotoxicity assay used to measure metabolic activity in viable cells. The assay is based on the reduction of the pink tetrazolium salt reagent 2-(2-methoxy-4-nitrophenyl)-3-(4-nitrophenyl)-5-(2,4-disulfophenyl)-2*H*-tetrazolium (WST-8) by metabolically active cells to a water-soluble orange formazan product. In this study, the WST-8 assay was used to evaluate the anticancer and cytotoxic effects of compounds **10**–**16**. For this purpose, A549, HeLa, and HDF-1 cells were seeded into 96-well plates at a density of 7 × 10^3^ cells per well and incubated for 24 h to allow for adherence. Following incubation, the cells were treated with the compounds at specified concentrations (6.25, 12.5, 25, 50, 100, 200 μM) for 48 h. Methotrexate (MTX) was used as a standard pharmacological anticancer positive control. All compounds were dissolved in dimethyl sulfoxide (DMSO), prior to treatment. In addition, 7% DMSO was included as an independent positive control due to its pronounced cytotoxic effects on the tested cell lines. Following the treatment period, 50 μL of a 10% WST-8 solution was added to each well and the plates were incubated at 37 °C in a 5% CO_2_ incubator for 4 h. The absorbance was then measured at 450 nm using a microplate reader.^25^ IC_50_ (half-maximal inhibitory concentration) values of the compounds were determined by comparing the absorbances of the treated and control groups.

$$\%\;\mathrm{viability}=\frac{\left[{\mathrm{Abs}}_{\mathrm{treated}}-{\mathrm{Abs}}_{\mathrm{WST‐8,cell‐free}}\right]}{\left[{\mathrm{Abs}}_{\mathrm{control}}-{\mathrm{Abs}}_{\mathrm{cell‐free}}\right]}\times 100\%$$

where Abs_treated_, Abs_control_, Abs_WST‑8,cell‑free_, and Abs_cell‑free_ are the absorbances of the cells treated with compounds, the control group cells, WST-8 in a cell-free environment, and the cell-free environment, respectively.

### 2.4 Antioxidant Assay

Test compounds were dissolved in DMSO and deionized water to prepare the stock solutions. A known antioxidant, such as ascorbic acid and β-carotene, was used as the positive control.

#### 2.4.1 CUPRAC Test

A 10 mM copper­(II) chloride (CuCl_2_) solution, 7.5 mM neocuproine solution, and 1000 mM ammonium acetate buffer (pH 7.0) were prepared. Test compounds and standards were prepared at various concentrations (6.25–200 μM). In each well of a 96-well microplate, 55 μL of the sample, 50 μL of CuCl_2_, 50 μL of neocuproine, and 50 μL of ammonium acetate buffer were added sequentially. The plate was incubated in the dark at room temperature for 30 min. After incubation, the absorbance was measured at 450 nm.^26^

#### 2.4.2 DPPH Test

A 1 mM DPPH solution was prepared by dissolving DPPH in methanol. Test compounds and standards were prepared at various concentrations (6.25–200 μM). In each well of a 96-well microplate, 50 μL of sample, 100 μL of ethanol, and 50 μL of DPPH solution were added sequentially. The plate was incubated in the dark at room temperature for 30 min. After incubation, the absorbance was measured at 517 nm.^27^

### 2.5 Antibacterial Activity

The antibacterial activities of the compounds were evaluated against *Acinetobacter baumannii* (ATCC BAA-1605), *Enterococcus faecalis* (ATCC 49452), *Enterococcus faecium* (ATCC 700221), *Pseudomonas aeruginosa* (ATCC 27853), *Escherichia coli* (ATCC BAA-2523), *Staphylococcus aureus* (ATCC 25923), and Methicillin-resistant *S. aureus* (MRSA) (ATCC 43300). Bacterial isolates were cultured on Mueller-Hinton Agar (MHA) and used in subsequent antibacterial assays.

#### 2.5.1 Agar Well Diffusion Assay

The bacterial inoculum was prepared to a 0.5 McFarland standard and spread-plated on MHA plates. Wells were created by puncturing the agar with sterile Koch tubes. Compounds prepared at a concentration of 200 μM were added to each well, and the plates were incubated at 37 °C for 24 h. The diameters of the inhibition zones were measured. All experiments were performed in triplicate with three technical replicates. Ampicillin (200 μM) was used as the positive control.^28^

#### 2.5.2 Broth Microdilution Method

To determine the minimum inhibitory concentration (MIC) of compounds showing antibacterial activity in the agar well diffusion test, a microdilution assay was performed. For this purpose, 100 μL of bacterial inoculum adjusted to a 0.5 McFarland standard was added to 96-well plates, followed by 100 μL of compounds prepared at concentrations of 6.25, 12.5, 25, 50, 100, and 200 μM in triplicate. All experiments were performed in triplicate with three technical replicates. The plates were incubated for 24 h. The lowest concentrations that prevent bacterial growth after incubation, indicated by the absence of visible turbidity, were recorded as the MIC.^29^

### 2.6 *In Silico* Analysis

Computational ADME (absorption, distribution, metabolism, and excretion) prediction of the synthesized compounds was performed by using SwissADME software. The drug-likeness and physicochemical properties of the synthesized compounds were evaluated.

### 2.7 Statistical Analysis

Statistical analyses of the data obtained in this study were performed using GraphPad Prism 5.00 software (GraphPad Software, La Jolla, CA, USA). Results are presented as mean ± standard deviation (STD), and statistical significance was determined using one-way ANOVA followed by Dunnett’s test, with *p* < 0.05 considered significant.

## 3 Results and Discussion

### 3.1 Chemistry

Various methods for the synthesis of aroyl thioureas have been reported in the literature. Among these, the approach utilizing chloroformate precursors is widely regarded as effective. For instance, a recent study by Pandey and co-workers demonstrated the synthesis of various *N*-aroyl, *N*′-substituted thioureas in good yields using this method.^24,30^ While the scaffolds reported by Pandey et al. primarily focused on *N*′-alkyl or *N*′-(un) substituted aryl moieties, our study was designed to introduce novelty by synthesizing a new series of *N*-(phenoxycarbonyl)-*N*′-(substituted-phenethyl) thioureas (**10**–**16**). This molecular design uniquely combines the phenoxycarbonyl group (derived from phenyl chloroformate) with various biologically significant phenethylamine structures (**3**–**9**), many of which are known for their roles as neurotransmitter precursors or receptor ligands in the central nervous system. This approach led to the synthesis of seven new aroyl thiourea derivatives (**10**–**16**), previously unreported in the literature, thus contributing to this field. It was also noted that Pandey and co-workers employed ammonium thiocyanate for the isothiocyanate intermediate synthesis. Based on our previous experience, we found that potassium thiocyanate, a significantly more cost-effective alternative, afforded comparable yields.

The synthesis pathway for the target aroyl thioureas (**10**–**16**) is summarized in Schemes 1 and 2. The key intermediate, phenoxycarbonyl isothiocyanate (**2**), was generated *in situ* from the reaction of phenyl chloroformate (**1**) with potassium thiocyanate. This intermediate (**2**) was then reacted with the corresponding substituted phenethylamines (**3**–**9**) to yield the final products (**10**–**16**).

**Scheme 2:**
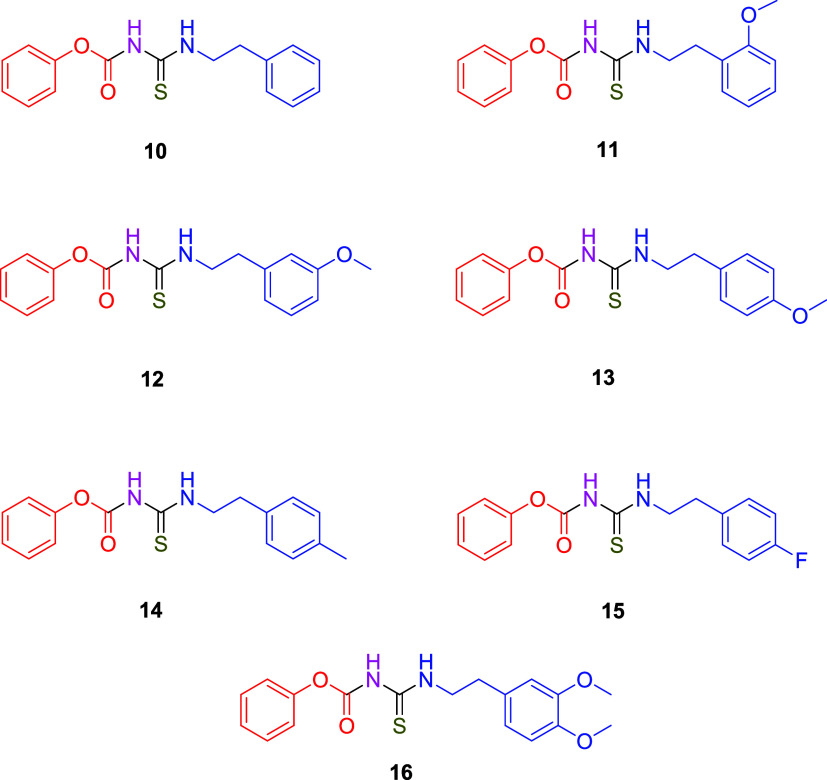
Synthesized Aroyl Thiourea Derivatives (**10**–**16**)

The ^1^H NMR spectra of the synthesized compounds displayed characteristic signals of the thiourea moiety. The −NH proton located between the carbonyl (C=O) and thiocarbonyl (C=S) groups appeared as a singlet at around 9.6 ppm, whereas the −NH proton adjacent to the thiocarbonyl group appeared as a singlet at around 8.5 ppm. In derivatives containing a methoxy group (−OCH_3_), the methoxy protons were observed as singlets at approximately 3.8 ppm, while methyl protons (−CH_3_) appeared as singlets near 2.3 ppm. The aliphatic −CH_2_ protons attached to the phenethylamine ring showed signals at ∼3.9 ppm (adjacent to −NH) and ∼2.8 ppm (proximal to the aromatic ring) (^1^H NMR spectra are provided in the Supporting Information).

The ^13^C NMR spectra exhibited characteristic thiocarbonyl (−CS) and carbonyl (−CO) signals at ∼179 and ∼159 ppm, respectively. Methoxy carbons appeared around 56.0 ppm, whereas methyl carbons were observed near 21.3 ppm. The aliphatic −CH_2_ carbons of the phenethylamine moiety resonated between 33.7 and 47.5 ppm, consistent with the proposed structures (^13^C NMR spectra are provided in the Supporting Information).

The FTIR spectra further confirmed the functional groups of the synthesized compounds. Broad to moderately intense bands at 3280–3032 cm^–1^ were assigned to N–H stretching vibrations of the thiourea moiety. Strong, sharp bands at 1724–1712 cm^–1^ corresponded to C=O stretching, confirming the presence of the carbonyl group. Bands in the range 1610–1454 cm^–1^ were attributed to N–H bending and C–N stretching vibrations, supporting the presence of both carbonyl and thiocarbonyl groups. Characteristic C=C vibrations of the benzene ring were observed between 1481 and 1328 cm^–1^, whereas the C=S stretching vibration appeared around 1332–1134 cm^–1^. In the 1151–1001 cm^–1^ region, C–N and C–O stretching and phenyl C–H bending vibrations were detected. The region 910–684 cm^–1^ corresponded to out-of-plane C–H bending in the aromatic ring, indicating the presence of the phenyl group. Finally, bands in the range 684–547 cm^–1^ were attributed to C–S stretching vibrations, characteristic of thiourea derivatives (FTIR spectra are provided in the Supporting Information).

### 3.2 Biological Evaluations

#### 3.2.1 Anticancer Activity

To evaluate the anticancer effects of the synthesized compounds (**10**–**16**) on A549 and HeLa cells, as well as their cytotoxicity toward normal HDF-1 cells, the cells were treated with increasing concentrations of the compounds (6.25–200 μM) for 48 h, and the WST-8 assay was performed. The results are summarized in Table 1 and are illustrated in Figures 2–4. The anticancer activities of the compounds were evaluated in A549 cells. Compound **14** exhibited the highest activity, with an IC_50_ value of 38.47 ± 0.73 μM (*p* < 0.001), and significantly reduced cell viability to below 50% at concentrations of 50–200 μM compared with the control group. Similarly, compound **15** (IC_50_ = 89.91 ± 0.23 μM; *p* < 0.001) reduced cell viability to below 50% at concentrations of 100 and 200 μM, while compound **16** (IC_50_ = 167.48 ± 0.29 μM; *p* < 0.001) showed a comparable effect only at 200 μM. Methotrexate (MTX), used as a positive control (IC_50_ = 48.73 ± 0.38 μM; *p* < 0.001), also reduced cell viability to below 50% at concentrations of 50–200 μM. Based on these results, compound **14** demonstrated greater anticancer activity than MTX. In addition, 7% DMSO significantly reduced cell viability below 50%. In contrast, compounds **10**–**13** caused a decrease in cell viability compared with the control but did not reduce viability below 50%, indicating moderate antiproliferative effects (Figure 2).

**Figure 2:**
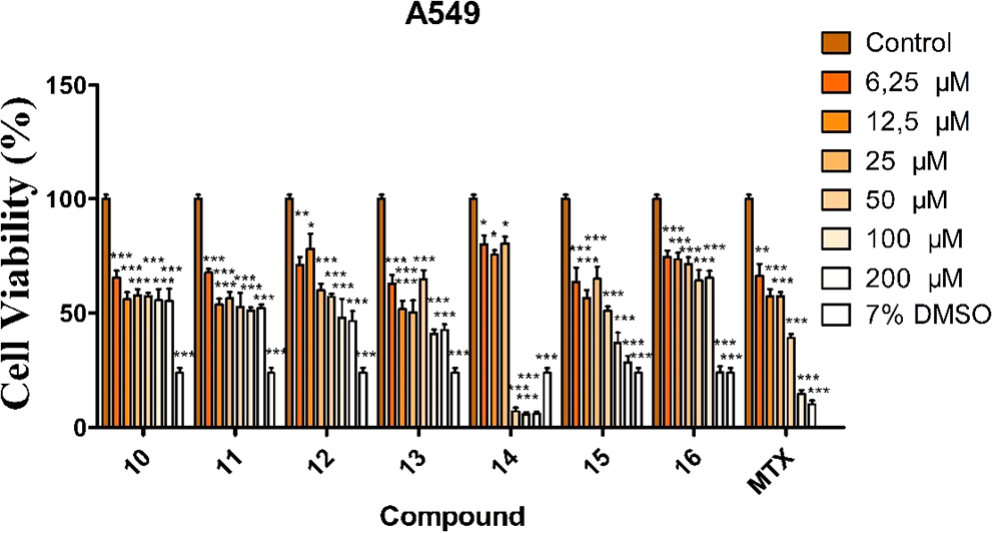
Anticancer activity of synthesized aroyl thiourea compounds in A549 cells (*p < 0.05, **p < 0.01, ***p < 0.001).

**Table 1:** IC_50_ Values of Compounds **10**–**16** in A549, HeLa, and HDF-1 Cells

	IC_50_ value (μM) ± STD		
compound	A549	HeLa	HDF-1
**10**	>200 ± 0.09	150.83 ± 0.31	>200 ± 0.03
**11**	>200 ± 0.05	>200 ± 0.11	>200 ± 0.06
**12**	>200 ± 0.19	>200 ± 0.07	>200 ± 0.29
**13**	>200 ± 0.10	>200 ± 0.13	>200 ± 0.09
**14**	38.47 ± 0.73	>200 ± 0.15	>200 ± 0.10
**15**	89.91 ± 0.23	>200 ± 0.06	>200 ± 0.11
**16**	167.48 ± 0.29	>200 ± 0.05	>200 ± 0.14
MTX	48.73 ± 0.38	123.23 ± 0.47	23.07 ± 0.19

The anticancer activities of the compounds were also evaluated in HeLa cells. Compound **10** exhibited limited activity, with an IC_50_ value of 150.83 ± 0.31 μM (*p* < 0.001), reducing cell viability to approximately 50% only at a concentration of 200 μM compared with the control group. In contrast, compounds **11**–**16** caused a decrease in cell viability but did not reduce the viability to 50%, indicating a lack of significant anticancer activity. Methotrexate (MTX), used as a positive control (IC_50_ = 123.23 ± 0.47 μM; *p* < 0.001), also reduced cell viability to approximately 50% at 200 μM. Similarly, 7% DMSO reduced cell viability to approximately 50% (Figure 3).

**Figure 3:**
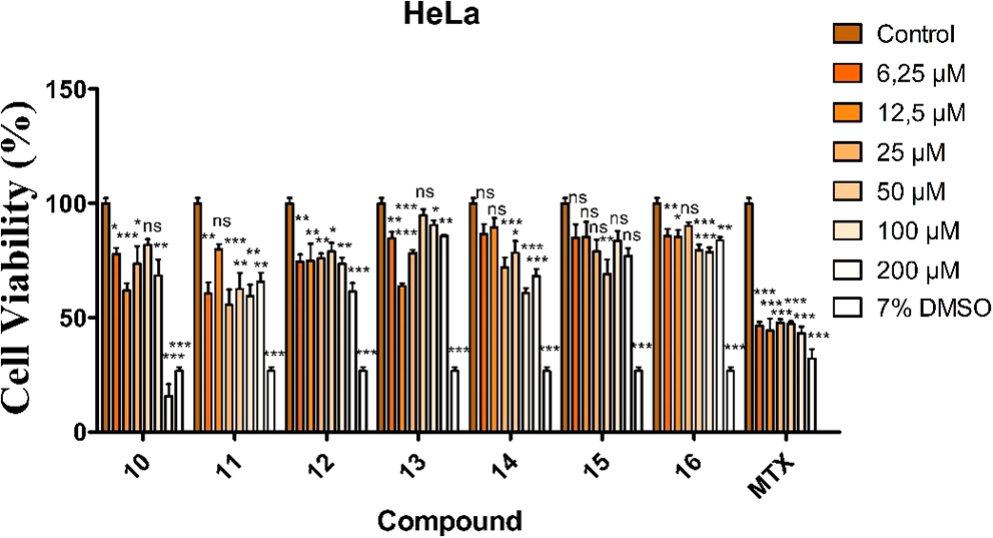
Anticancer activity of synthesized aroyl thiourea compounds on the HeLa cells (*p* > 0.05, *p < 0.05, **p < 0.01, ***p < 0.001).

In HDF-1 cells, used to assess the cytotoxic effects of the compounds on a healthy cell line, none of the synthesized compounds exerted significant cytotoxicity, and cell viability remained above 50% at all tested concentrations. Methotrexate (MTX), used as a positive control, exhibited an IC_50_ value of 23.07 ± 0.19 μM (*p* < 0.001) and reduced cell viability to below 50% at concentrations ranging from 25 to 200 μM compared with the control group. In contrast, the synthesized compounds showed lower cytotoxic effects than MTX. In addition, 7% DMSO (*p* < 0.001) also reduced cell viability to below 50% (Figure 4).

**Figure 4:**
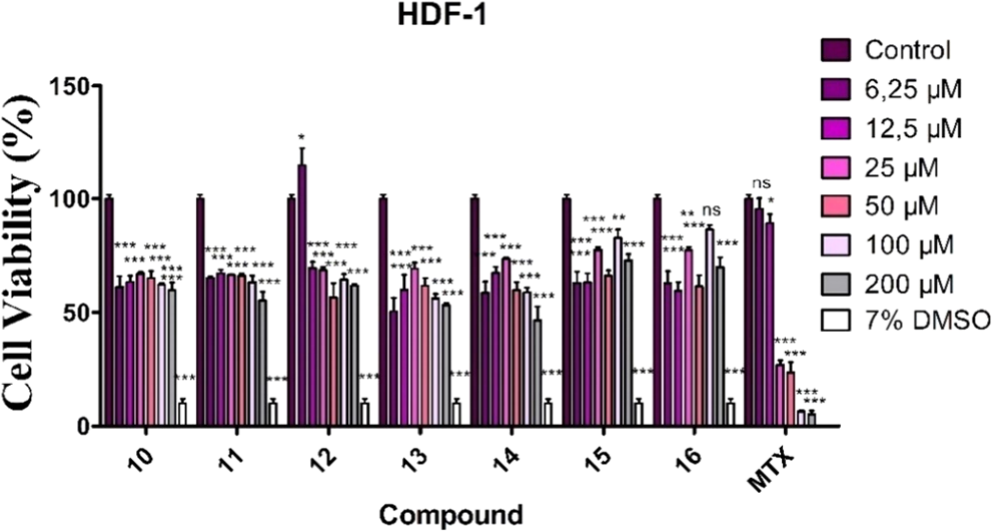
Cytotoxic effects of synthesized aroyl thiourea compounds on the HDF-1 cells (*p* > 0.05, **p < 0.01, ***p < 0.001).

When the structure–activity relationships of the compounds were examined, compound **14**, which exhibited the most notable activity in A549 cells, was found to contain a methyl group at the para position of the phenethylamine ring. In HeLa cells, the most notable activity was observed for compound **10**, which lacks any substitution on the phenylethylamine ring. In the HDF-1 cell line, compound **12**, possessing a methoxy group at the meta position, displayed the lowest cytotoxic effect among all synthesized derivatives.

It has also been reported in the literature that aroyl thiourea derivatives and phenethylamine-based thioureas possess anticancer activity.^31^ Another study demonstrated that the synthesized compounds exhibited low toxicity toward the HDF-1 cell line, which represents a healthy cell line.^23^ However, it is thought that the absence of a significant dose-dependent increase or decrease in the viability of A549, HeLa, and HDF-1 cells observed in our study may be attributed to the cytostatic and cytotoxic effects of the compounds. In the literature, there are reports indicating that the lack of clear dose-dependent variation in cytotoxicity tests may result from the cytostatic or cytotoxic nature of the tested substances.^32,33^

#### 3.2.2 Antioxidant Activity

In the CUPRAC assay, which is based on the reduction of Cu^2+^ to Cu^+^, the radical scavenging activities of the synthesized compounds were evaluated at various concentrations (6.25 and 200 μM), using ascorbic acid and β-carotene as reference antioxidants. The order of radical scavenging activity was found tobe ascorbic acid > **10** > β-carotene > **16** > **13** > **12** > **11** > **15** > **14**. The IC_50_ values of the synthesized and reference compounds were determined as follows: ascorbic acid, 48.7 ± 1.17 μM; compound **10**, 90.17 ± 0.15 μM; β-carotene, 120.06 ± 0.28 μM; compound **16**, 168.59 ± 0.28 μM; compound **13**, 176.55 ± 0.30 μM; compound **12**, 178.50 ± 0.22 μM; compound **11**, 179.15 ± 0.44 μM; compound **15**, 193.41 ± 0.53 μM; compound **14**, 195.08 ± 0.23 μM. Among the synthesized derivatives, compound **10** exhibited the most notable antioxidant activity, showing a higher radical scavenging potential than β-carotene (Table 2 and Figure 5).

**Figure 5:**
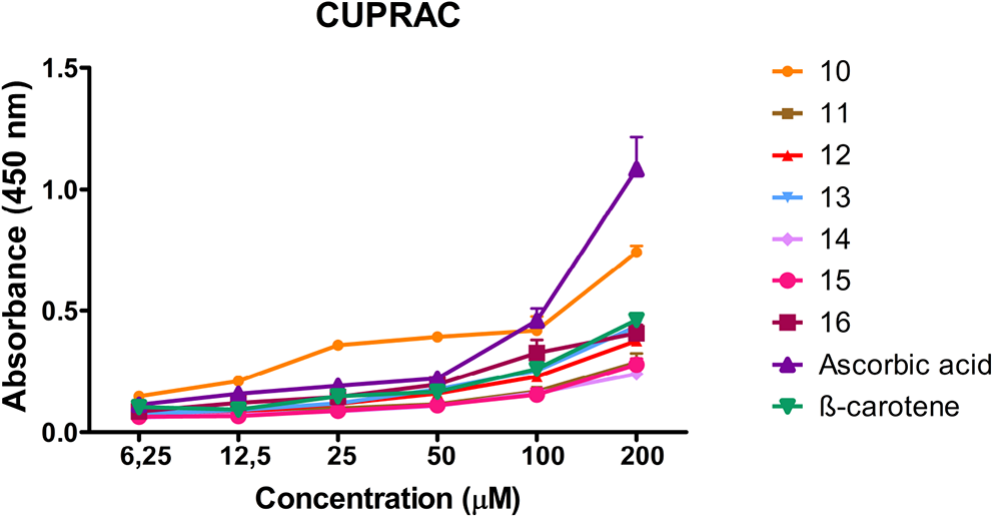
CUPRAC radical scavenging activity of compounds **10**–**16** and reference antioxidants at different concentrations.

**Table 2:** CUPRAC and DPPH IC_50_ Values for Compounds **10**–**16**

	IC_50_ value (μM) ± STD	
compound	CUPRAC	DPPH
**10**	90.17 ± 0.15	202.63 ± 4.54
**11**	179.15 ± 0.44	242.83 ± 0.24
**12**	178.50 ± 0.22	235.78 ± 0.08
**13**	176.55 ± 0.30	213.72 ± 7.91
**14**	195.08 ± 0.23	246.63 ± 0.20
**15**	193.41 ± 0.53	252.68 ± 1.03
**16**	168.59 ± 0.28	249.30 ± 0.21
ascorbic acid	48.7 ± 1.17	70.72 ± 0.50
β-carotene	120.06 ± 0.28	90.22 ± 5.09

The DPPH radical scavenging activities of the synthesized compounds were evaluated at various concentrations (6.25–200 μM), using ascorbic acid and β-carotene as reference antioxidants. The order of radical scavenging activity was found to be ascorbic acid > β-carotene > **10** > **13** > **12** > **11** > **14** > **16** > **15**. The IC_50_ values of the synthesized and reference compounds were calculated as follows: ascorbic acid, 70.72 ± 0.50 μM; β-carotene, 90.22 ± 5.09 μM; compound **10**, 202.63 ± 4.54 μM; compound **13**, 213.72 ± 7.91 μM; compound **12**, 235.78 ± 0.08 μM; compound **11**, 242.83 ± 0.24 μM; compound **14**, 246.63 ± 0.20 μM; compound **16**, 249.30 ± 0.21 μM; compound **15**, 252.68 ± 1.03 μM. Overall, the synthesized compounds exhibited relatively low antioxidant activity at all tested concentrations compared with the reference antioxidants (Table 2 and Figure 6).

**Figure 6:**
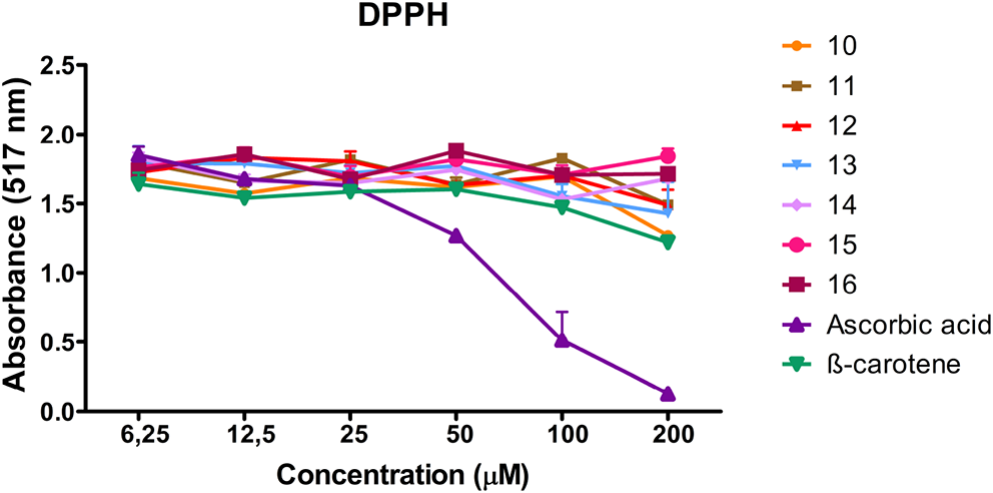
DPPH radical scavenging activity of compounds **10**–**16** and reference antioxidants at different concentrations.

When the structure–activity relationships (SAR) of the synthesized compounds were analyzed, compound **10**—lacking any substituent on the phenethylamine ring—demonstrated the most notable antioxidant activity in both the CUPRAC and DPPH assays. Previous reports on aroyl thiourea derivatives have indicated that while some compounds display DPPH radical scavenging activity, others show limited or negligible antioxidant potential.^2^ Furthermore, another study using the CUPRAC method reported that phenethylamine-based derivatives exhibited notable antioxidant activity.^23^ These findings from the literature are in agreement with the results obtained in the present study.

#### 3.2.3 Antibacterial Activity

The antibacterial activities of the synthesized thiourea derivatives were evaluated against seven different bacterial isolates (three Gram-negative and four Gram-positive), known to be particularly resistant to multiple drugs, using agar well diffusion and microdilution methods. Among the bacterial isolates used in the study, all except *E. faecalis* were resistant to the standard antibiotic ampicillin; *E. faecalis* showed slight sensitivity, with an inhibition zone of 1.16 cm. Moreover, the synthesized compounds demonstrated varying degrees of antibacterial activity against the tested isolates. The results of the antibacterial activity and MIC values are presented in Tables 3 and 4, respectively.

**Table 3:** Antibacterial Activities of Compounds **10**–**16**

	activity^a^							
bacterial strain	**10**	**11**	**12**	**13**	**14**	**15**	**16**	ampicillin
MRSA ATCC 43300	1.2 ± 0.29	1.0 ± 0.81	1.26 ± 0.48	1.36 ± 0.14	1.15 ± 0.43	1.05 ± 0.21	1.15 ± 0.12	
*A. baumannii* ATCC BAA 1605	0.9 ± 0.34	1.0 ± 0.49	1.55 ± 0.77	1.0 ± 0.52	1.0 ± 0.18	1.35 ± 0.63	0.95 ± 0.19	
*E. faecalis* ATCC 49452	1.43 ± 0.5	1.63 ± 0.97	1.65 ± 0.58	1.6 ± 0.33	2 ± 0.62	1.83 ± 0.25	1.5 ± 0.62	1.16 ± 0.36
*E. faecium* ATCC 700221	1.9 ± 0.18	1.85 ± 0.8	1.6 ± 0.29	1.85 ± 0.6	1.66 ± 0.11	1.56 ± 0.92	1.5 ± 0.44	
*E. coli* ATCC 2523	1.03 ± 0.72	1.0 ± 0.26	1.16 ± 0.36	0.86 ± 0.21	1.53 ± 0.49	1.13 ± 0.41	1.1 ± 0.53	
*P. aureginosa* ATCC 27853	1.15 ± 0.91	1.25 ± 0.81	2 ± 0.65	0.85 ± 0.18	1.0 ± 0.73	1.1 ± 0.76	1.15 ± 0.74	
*S. aureus* ATCC 25922	1.03 ± 0.33	1.05 ± 0.63	1.1 ± 1.02	1.13 ± 0.46	1.1 ± 0.38	1.06 ± 0.83	1.2 ± 0.29	

a Zone diameter (cm).

**Table 4:** Minimum Inhibitory Concentration (MIC) of Compounds **10**–**16**

	MIC (μm)							
bacterial strain	**10**	**11**	**12**	**13**	**14**	**15**	**16**	ampicillin
MRSA ATCC 43300	12.5	12.5	6.25	12.5	12.5	12.5	100	500
*A. baumanni* ATCC BAA 1605	12.5	12.5	6.25	12.5	25	12.5	25	500
*E. faecalis* ATCC 49452	12.5	12.5	25	12.5	25	6.25	12.5	125
*E. faecium* ATCC 700221	12.5	25	6.25	6.25	6.25	6.25	25	125
*E. coli* ATCC 2523	25	25	12.5	25	25	25	25	250
*P. aureginosa* ATCC 27853	50	50	25	100	100	50	100	500
*S. aureus* ATCC 25922	50	50	50	12.5	25	12.5	25	125

Among the tested compounds, compound **12** exhibited higher antibacterial activity than ampicillin against all bacterial isolates. Structure–activity relationship analysis revealed that the most effective compound, thiourea **12**, contained a 3-methoxy phenethylamine ring, while the second most active compound, **15**, contained a 4-fluoro phenethylamine ring.

Among the synthesized compounds, compounds **11**, **12**, **13**, **14**, and **16** possess electron-donating characteristics due to the presence of methoxy and methyl substituents in their structures, whereas compound **15**, which contains a fluorine substituent, exhibits electron-withdrawing properties. Electron-donating groups increase the electron density of compounds, while electron-withdrawing groups decrease it, which can, in turn, influence their chemical reactivity. Although some of the synthesized compounds demonstrated notable biological activity, others exhibited a relatively low activity. These differences in biological activity are likely attributable not only to the presence of electron-donating or -withdrawing groups but also to factors such as binding orientation, steric effects, and intramolecular interactions.

Previous studies have reported that methoxy substituents enhance antibacterial activity and that compounds bearing methoxy groups at the *ortho*, *meta*, or *para* positions inhibit the growth of many microorganisms.^34^ Consistent with these findings, compound **12**, which contains a methoxy group at the *meta* position, showed strong antibacterial activity against the tested isolates. In addition, the presence of a fluorine atom is known to enhance biological activity due to its strong electron-withdrawing nature.^35^ Accordingly, compound **15**, which contains a fluorine atom in its structure, also exhibited a high antibacterial activity.

#### 3.2.4 *In Silico* ADMET Prediction

The development of effective therapeutic drugs is a costly and time-consuming process that requires substantial effort. Ideally, drug candidates should exhibit maximal efficacy with minimal toxicity. However, a large proportion of drug candidates fail due to poor efficacy or undesirable pharmacological effects, and many failures in drug development are primarily associated with high toxicity.^36^ Considering ADME/Tox parameters (absorption, distribution, metabolism, elimination, and toxicity) during the early stages of drug design enhances efficiency and facilitates the identification of active compounds with fewer side effects and lower toxicity. ADMET data enable the simultaneous evaluation of multiple pharmacokinetic parameters, thereby supporting the selection of compounds with optimal drug-like properties.^37^ In the present study, *in silico* analyses of the synthesized aroyl thiourea derivatives (**10**–**16**) were performed. The lipophilicity (LIPO), size (SIZE), polarity (POLAR), insolubility (INSOLU), flexibility (FLEX), and saturation (INSATU) of the compounds were first predicted by using the SwissADME software tool. Drug-likeness and bioavailability were subsequently evaluated using bioavailability radar plots (Figure 7). The radar plots display optimal descriptor regions within a pink hexagon along each axis; compounds that fall within this region are considered drug-like. The topological polar surface area (TPSA) values of all synthesized aroyl thiourea derivatives ranged from 82.45 to 100.91 Å^2^, which lies within the optimal range (20–130 Å^2^), suggesting high oral bioavailability. According to the radar plots, the physicochemical parameters of compounds **10**, **11**, **12**, **13**, **14**, and **15**, including lipophilicity, size, polarity, solubility, and flexibility, were within the optimal pink region, while their saturation values were slightly outside this range. Compound **16** exhibited optimal lipophilicity, size, polarity, and solubility; however, its flexibility and saturation parameters fell outside the ideal range.

**Figure 7:**
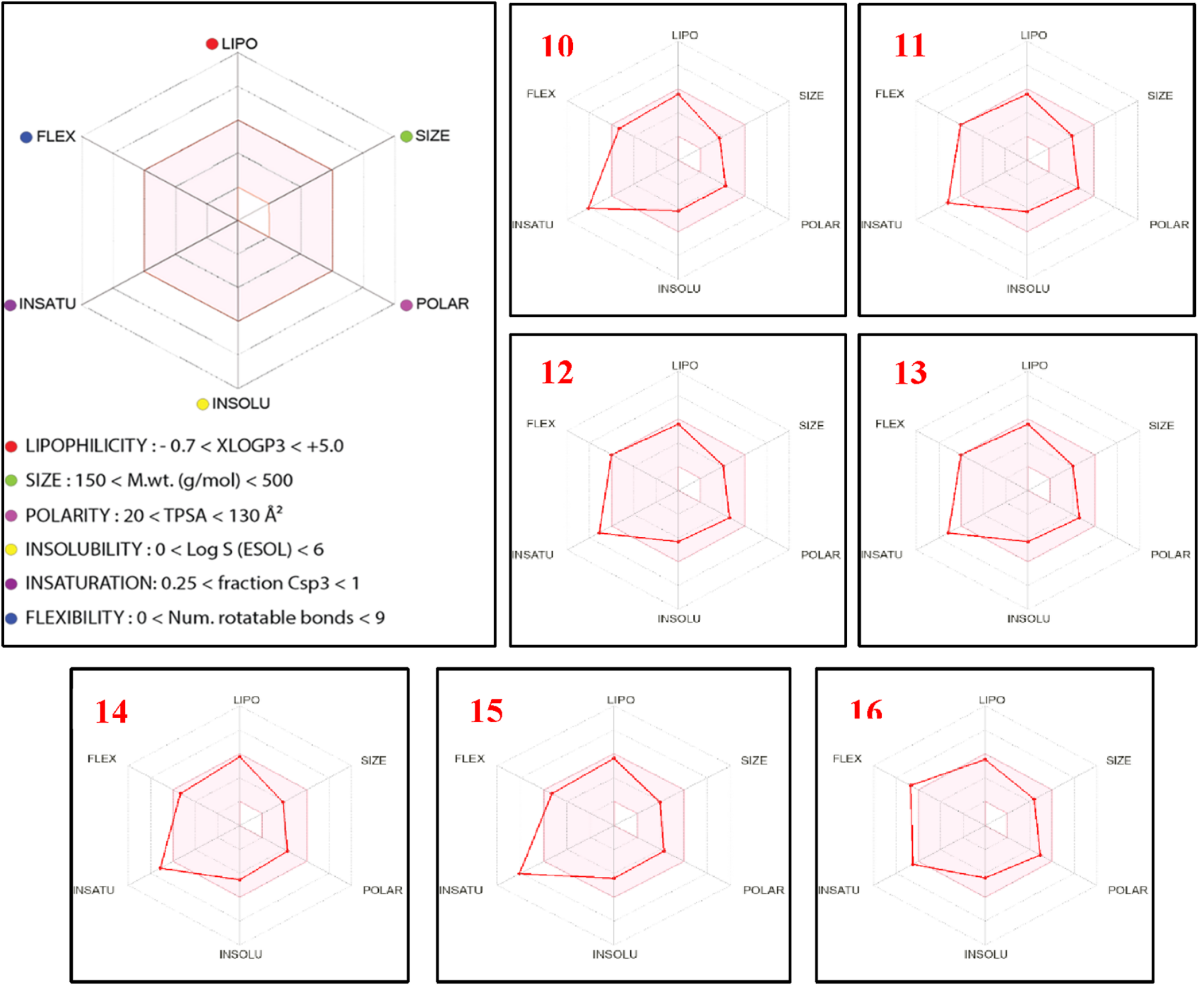
Bioavailability radar plots of compounds **10**–**16** based on their physicochemical properties.

Lipophilicity refers to the ability of a molecule to dissolve in lipid or lipid-like environments. For a drug to exhibit high bioavailability, it must effectively traverse biological membranes such as those of the gastrointestinal tract, the blood–brain barrier, and the skin. This requires an appropriate balance between aqueous and lipid solubility. The XLOGP3 (Log *P*_o/w_) values of the synthesized compounds ranged from 4.16 to 4.58, indicating relatively high lipophilicity. This property suggests that the compounds can readily cross lipid-rich biological barriers. The predicted water solubility values ranged from −4.26 to −4.55, indicating that the compounds also possess favorable aqueous solubility. Together, these lipophilic and hydrophilic characteristics are advantageous for efficient transport and distribution within the body.

Cytochrome P450 (CYP) enzymes constitute a superfamily that catalyzes diverse biochemical reactions and plays crucial roles in drug metabolism, toxin detoxification, and hormone synthesis. These enzymes are abundantly expressed in the liver and are also found in other tissues such as the intestines, kidneys, and lungs. The roles of CYPs in drug metabolism are particularly important, as they can alter the pharmacological effects and interactions of drugs by activating or deactivating them.^38^ Among human CYP enzymes, CYP1A2, CYP3A4, CYP2C9, CYP2C19, and CYP2D6 are the five most significant, collectively responsible for the biotransformation of the majority of therapeutic compounds. In the present study, all synthesized compounds were predicted to act as inhibitors of CYP1A2, CYP2C9, and CYP2C19, suggesting their potential to reduce the enzymatic activity of these isoforms. Compound **10** was also identified as a CYP2D6 inhibitor, indicating its ability to interfere with the activity of this enzyme, whereas compounds **11**–**16** were not predicted to inhibit CYP2D6. Furthermore, none of the synthesized derivatives were identified as CYP3A4 inhibitors—the most prevalent enzyme in human drug metabolism. The predicted Log *Kp* values, which indicate skin permeability, ranged from −4.97 to −5.55 cm/s, suggesting moderate permeability across the skin barrier (Table 5).

**Table 5:** Physicochemical Properties and Drug-likeness/drug Potential of Compounds **10**–**16**

property	rule	**10**	**11**	**12**	**13**	**14**	**15**	**16**
MW	<500	300.38	330.40	330.40	330.40	314.40	318.37	360.43
rotatable bonds	≤9	8	9	9	9	8	8	10
H-bond donors	≤5	2	2	2	2	2	2	2
H-bond acceptors	≤10	2	3	3	3	2	3	4
molar refractivity		85.83	92.32	92.32	92.32	90.79	85.78	98.81
TPSA (Å^2^)	<130	82.45	91.68	91.68	91.68	82.45	82.45	100.91
log *P*_**o** */* **w**_ (XLOGP3)	≤5	4.22	4.19	4.19	4.19	4.58	4.32	4.16
log *S* (ESOL)		–4.26	–4.32	–4.32	–4.32	–4.55	–4.41	–4.39
GI absorption		high	high	high	high	high	high	high
BBB permeant		no	no	no	no	no	No	no
Pgp substrate		no	no	no	no	no	No	no
CYP1A2 inhibitor		yes	yes	yes	yes	yes	Yes	yes
CYP2C19 inhibitor		yes	yes	yes	yes	yes	Yes	yes
CYP2C9 inhibitor		yes	yes	yes	yes	yes	Yes	yes
CYP2D6 inhibitor		yes	no	no	no	no	No	no
CYP3A4 inhibitor		no	no	no	no	no	No	no
log *Kp*(cm/s)		–5.14	–5.34	–5.34	–5.34	–4.97	–5.17	–5.55
Lipinski violation (LV)	≤1	0	0	0	0	0	0	0
bioavailability score		0.55	0.55	0.55	0.55	0.55	0.55	0.55
PAINS alerts		0	0	0	0	0	0	0
brenk alerts		1	1	1	1	1	1	1
synthetic accessibility		2.36	2.56	2.59	2.52	2.45	2.43	2.78

Parameters such as absorption, hydrophilic–lipophilic balance, polarity, molecular size, and molecular weight significantly influence a drug candidate’s ability to enter systemic circulation through the blood and lymphatic systems. The boiled-egg diagram (Figure 8) illustrates these properties by simultaneously predicting two key ADME characteristics: passive gastrointestinal absorption (HIA) and blood–brain barrier (BBB) permeability. This descriptive model utilizes two physicochemical parameters—WLOGP and TPSA—to estimate drug disposition.^39^ In the boiled-egg plot, three regions are defined: gray, white, and yellow. The outer gray zone represents compounds with minimal gastrointestinal absorption and limited BBB penetration. The middle white zone corresponds to molecules with high gastrointestinal absorption but poor BBB permeability, while the inner yellow zone indicates compounds predicted to exhibit both high gastrointestinal absorption and BBB penetration.^40^ In the diagram, blue and red dots denote compounds interacting with the P-glycoprotein (PGP) efflux transporter, which influences drug bioavailability and tissue distribution by actively exporting molecules out of the cells. Compounds identified as PGP-positive (PGP^+^) are substrates for this transporter, resulting in reduced intracellular accumulation and potentially limiting therapeutic efficacy—particularly relevant in the context of multidrug resistance and cancer therapy. Conversely, PGP-negative (PGP^–^) compounds are not actively effluxed, enabling more efficient tissue penetration and potentially enhancing drug efficacy while minimizing side effects.^41,42^ Analysis of the boiled-egg diagrams for the synthesized compounds revealed that all derivatives exhibited high gastrointestinal absorption and were not substrates of P-glycoprotein (PGP^–^). However, none of the compounds were predicted to cross the blood–brain barrier (Figure 8 and Table 5).

**Figure 8:**
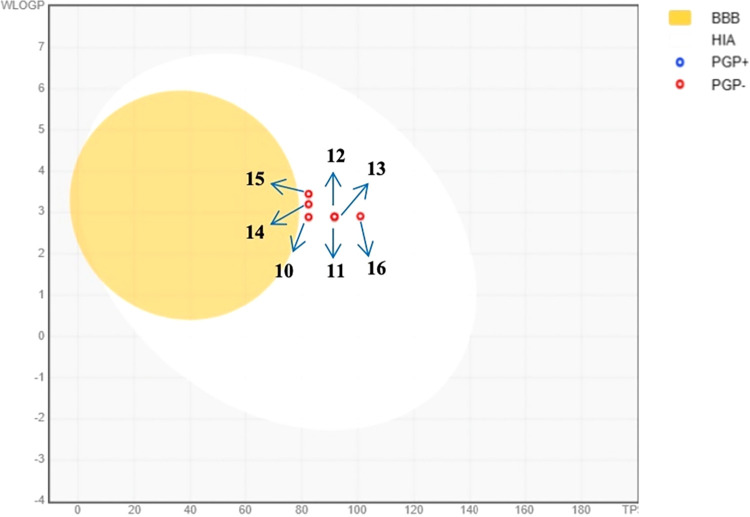
BOILED-egg model of synthesized aroyl thiourea compounds **10**–**16**.

## 4 Conclusion

In this study, seven phenethylamine-based aroyl thiourea derivatives (**10**–**16**) were successfully synthesized and evaluated for their biological activities. The anticancer and cytotoxic effects of the synthesized compounds were evaluated, revealing that compound **14** exhibited the highest anticancer activity in A549 cells, while compound **10** showed the greatest activity in HeLa cells; however, neither compound displayed significant cytotoxicity toward the normal HDF-1 cell line. Antioxidant activities assessed using CUPRAC and DPPH assays demonstrated that compound **10** possessed the strongest antioxidant potential in both systems. Antibacterial screening, conducted through agar well diffusion and microdilution methods, indicated that compounds **12** and **15** displayed notable antibacterial activity. In addition, the physicochemical properties and drug-likeness of all synthesized compounds were predicted using the SwissADME software tool. The compounds generally exhibited acceptable physicochemical properties and showed promising characteristics in terms of drug-likeness.

## Supplementary Material



## Notes

^1^H NMR spectrum, ^13^C NMR spectrum, and FTIR transmittance spectrum of all compounds (PDF)
