# Supplementary material for: Novel Phenethylamine-Based Aroyl Thiourea Derivatives: Design, Synthesis, and Multibiological Evaluation
Source: ACS Omega. 2026 Mar 17;11(12):19063–73. doi: 10.1021/acsomega.5c11616 (PMC13044656; doi:10.1021/acsomega.5c11616)
Supplement: Supplementary file 1 [file ao5c11616_si_001.pdf]

Novel Phenethylamine-Based Aroyl Thiourea Derivatives: Design, Synthesis, and Multi-Biological Evaluation

Bunyamin Ozgeris [a§], Elif Aksakal [b§], and Arzu Gormez [c*]

[a] *Depar^tm^ent of Basic Sciences, Faculty of Science, Erzurum Technical University, Erzurum, 25050, Turkey*

[b] *Department of Molecular Biology and Genetics, Faculty of Science, Erzurum Technical University, Erzurum, 25050, Turkey*

[c] *Department of Biology, Faculty of Science, Dokuz Eylul University, Izmir, 35390, Turkey*

* Email: arzu.gormez@deu.edu.tr

[§] *B.O. and E.A. contributed equally to this work*

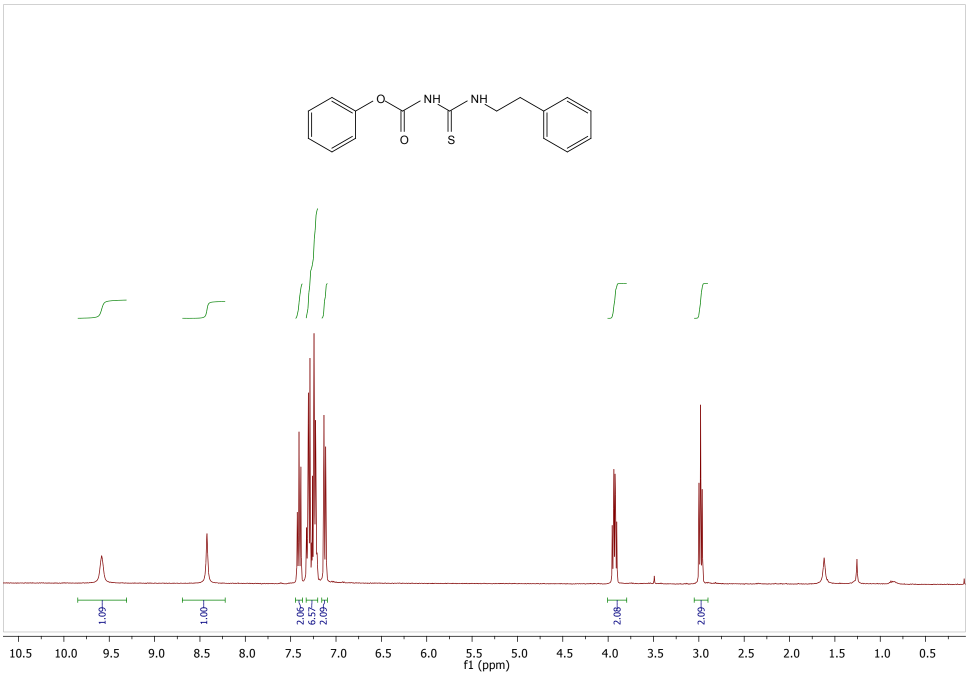

Figure S1. ^{1}H NMR spectrum of N-phenethyl-N'-(phenoxycarbonyl) thiocarbamide **(10)**

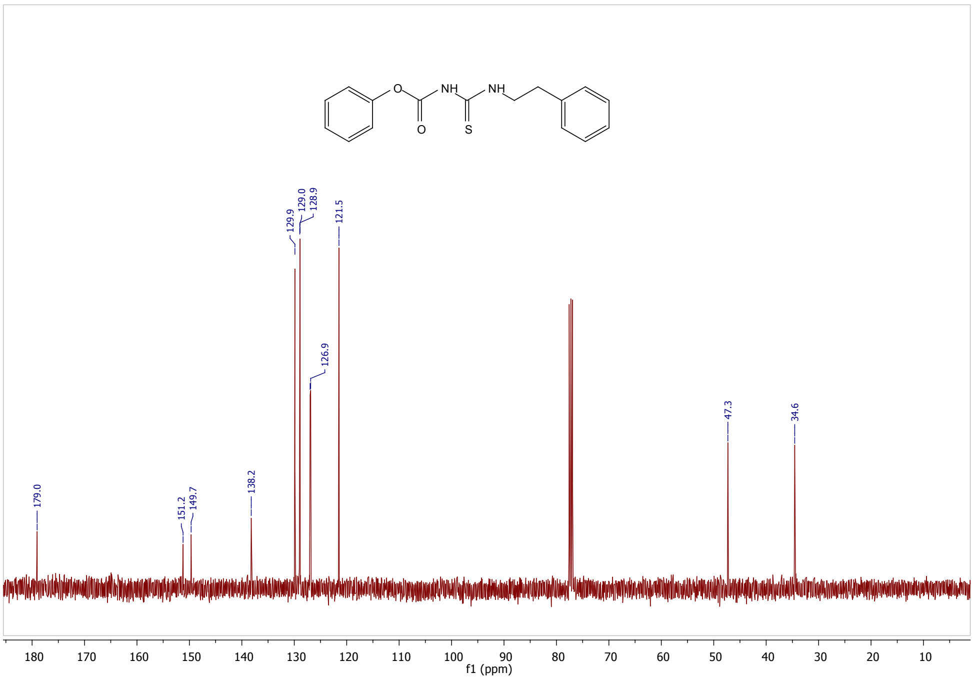

Figure S2. ^{13}C NMR spectrum of N-phenethyl-N'-(phenoxycarbonyl) thiocarbamide **(10)**

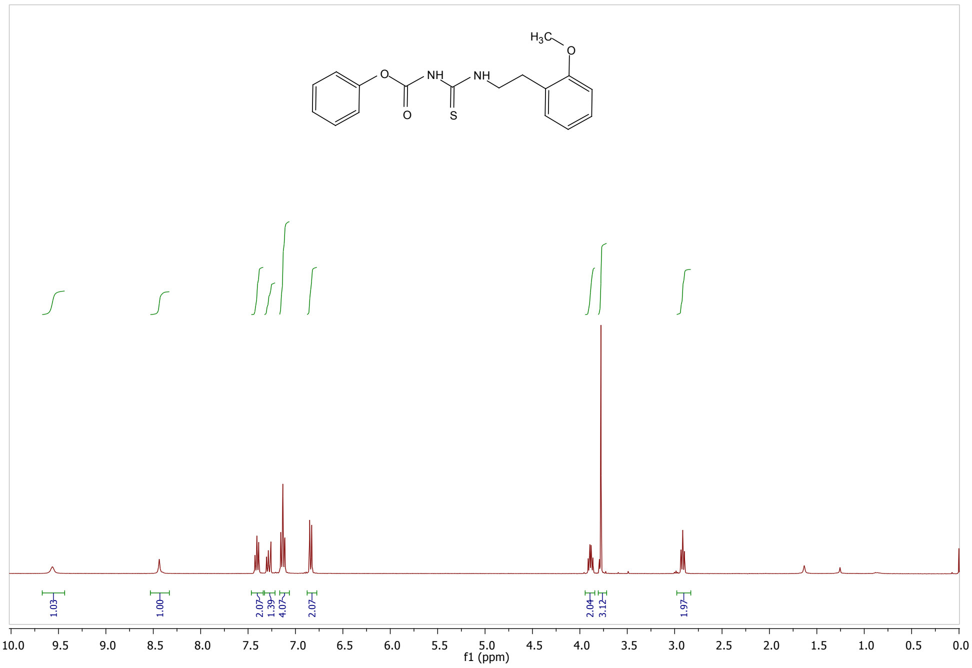

Figure S3. ^{1}H NMR spectrum of N-(2-methoxyphenethyl)-N'-(phenoxycarbonyl) thiocarbamide **(11)**

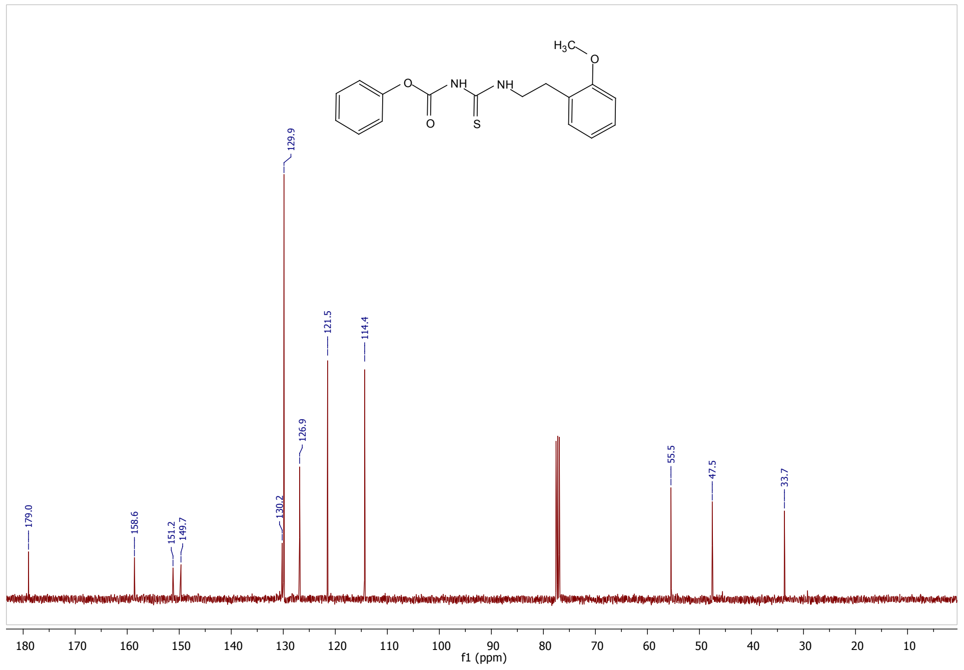

Figure S4. ^{13}C NMR spectrum of N-(2-methoxyphenethyl)-N'-(phenoxycarbonyl) thiocarbamide **(11)**

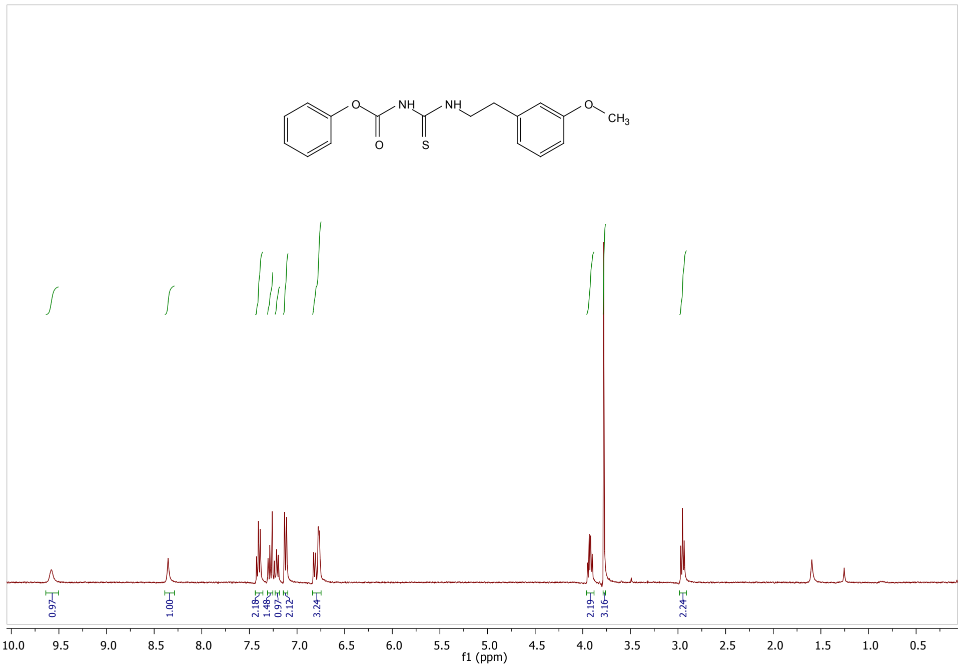

Figure S5. ^{1}H NMR spectrum of N-(3-methoxyphenethyl)-N'-(phenoxycarbonyl) thiocarbamide **(12)**

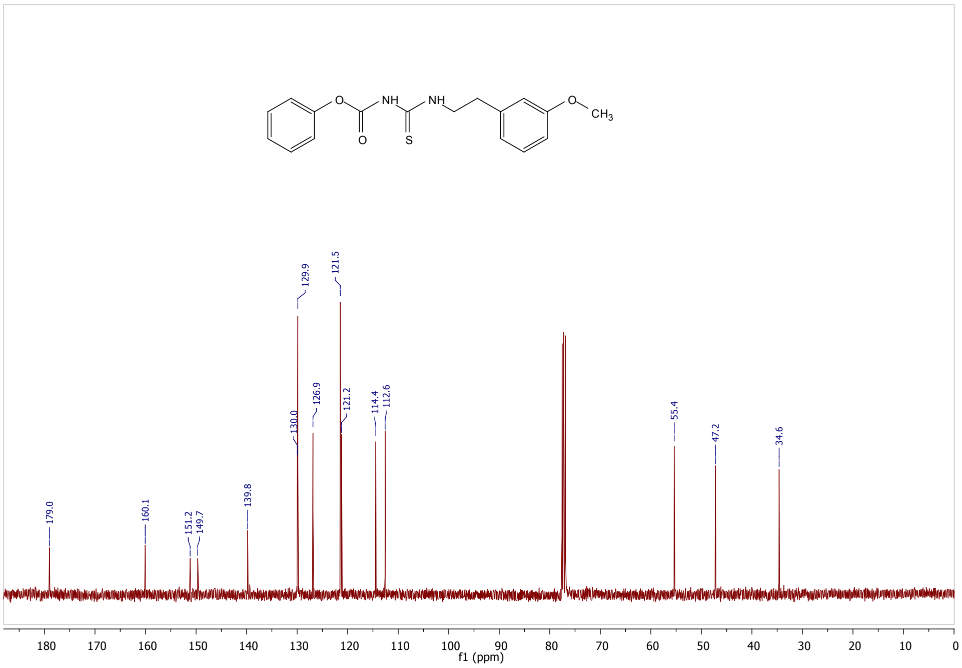

Figure S6. ^{13}C NMR spectrum of N-(3-methoxyphenethyl)-N'-(phenoxycarbonyl) thiocarbamide **(12)**

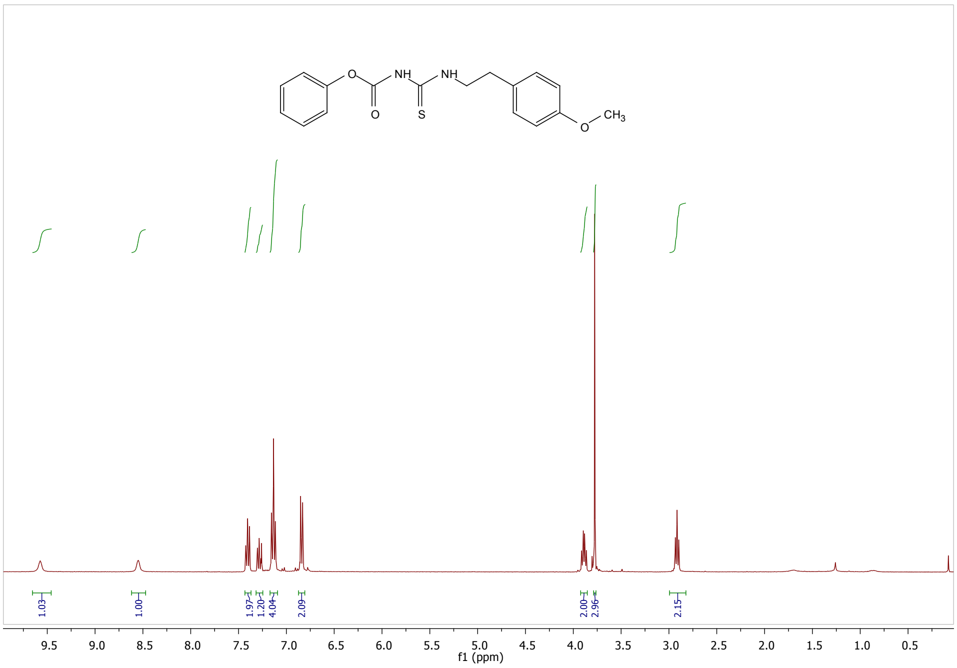

Figure S7. ^{1}H NMR spectrum of N-(4-methoxyphenethyl)-N'-(phenoxycarbonyl) thiocarbamide **(13)**

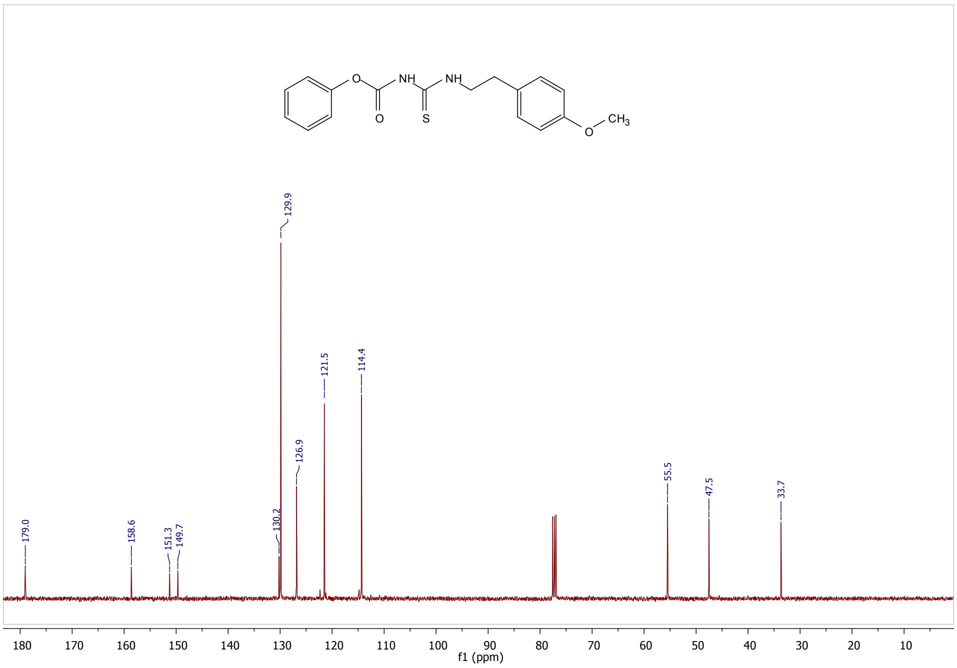

Figure S8. ^{13}C NMR spectrum of N-(4-methoxyphenethyl)-N'-(phenoxycarbonyl) thiocarbamide **(13)**

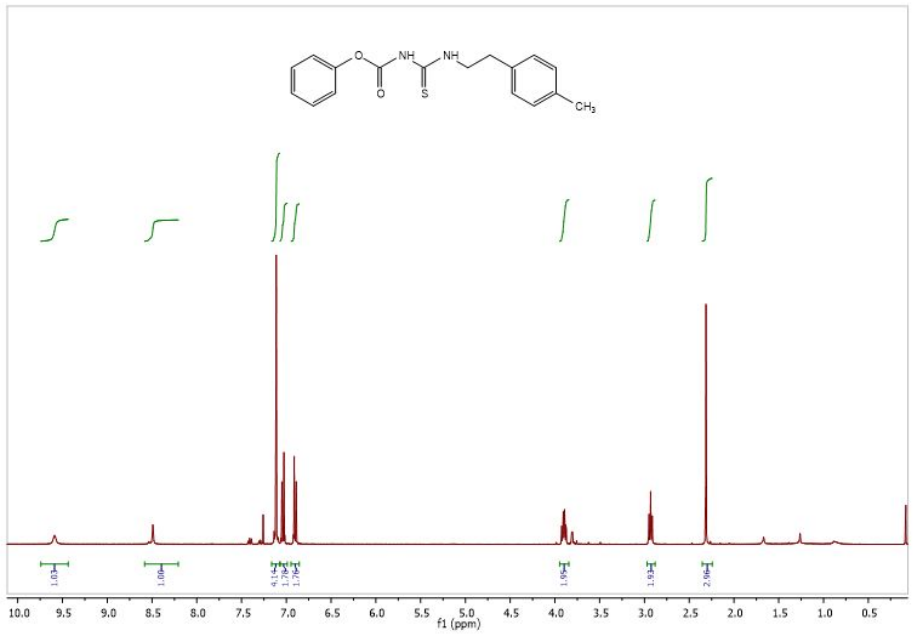

Figure S9. ^{1}H NMR spectrum of N-(4-methylphenethyl)-N'-(phenoxycarbonyl) thiocarbamide **(14)**

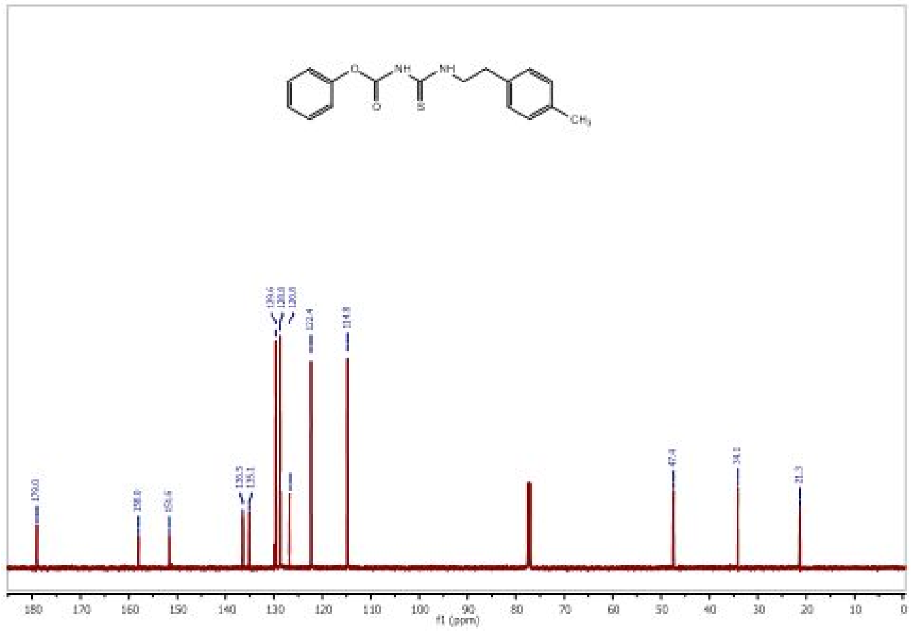

Figure S10. ^{13}C NMR spectrum of N-(4-methylphenethyl)-N'-(phenoxycarbonyl) thiocarbamide **(14)**

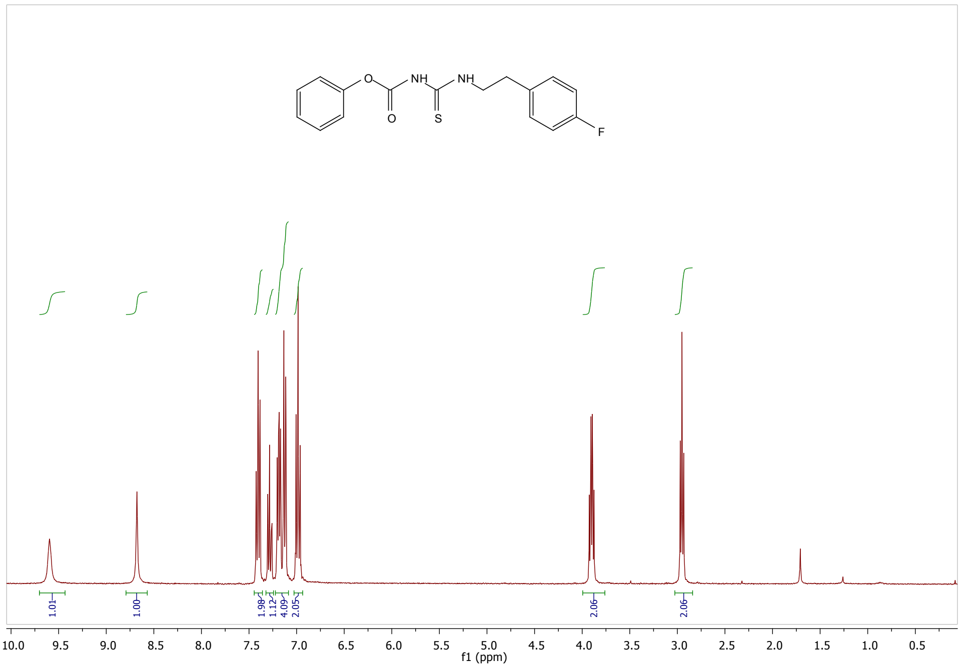

Figure S11. ^{1}H NMR spectrum of N-(4-fluorophenethyl)-N'-(phenoxycarbonyl) thiocarbamide **(15)**

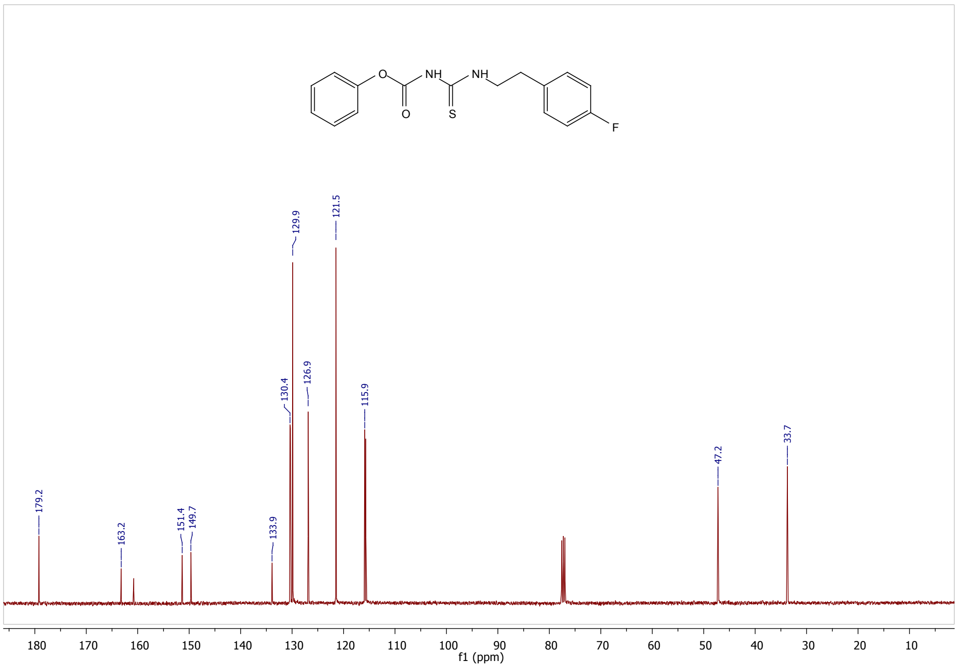

Figure S12. ^{13}C NMR spectrum of N-(4-fluorophenethyl)-N'-(phenoxycarbonyl) thiocarbamide **(15)**

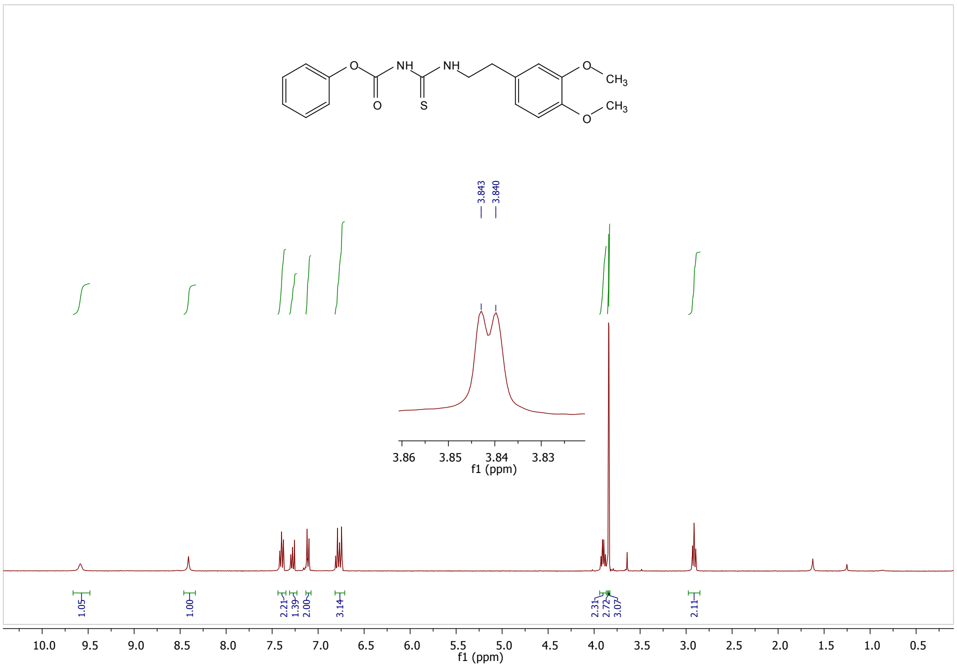

Figure S13. ^{1}H NMR spectrum of N-(3,4-dimethoxyphenethyl)-N'-(phenoxycarbonyl) thiocarbamide **(16)**

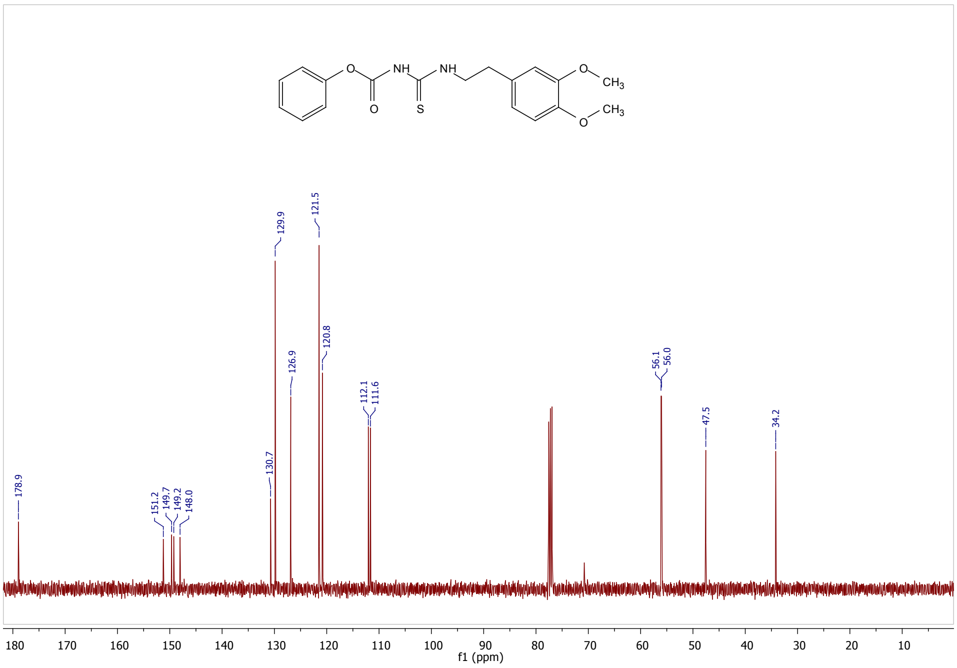

Figure S14. ^{13}C NMR spectrum of N-(3,4-dimethoxyphenethyl)-N'-(phenoxycarbonyl) thiocarbamide **(16)**

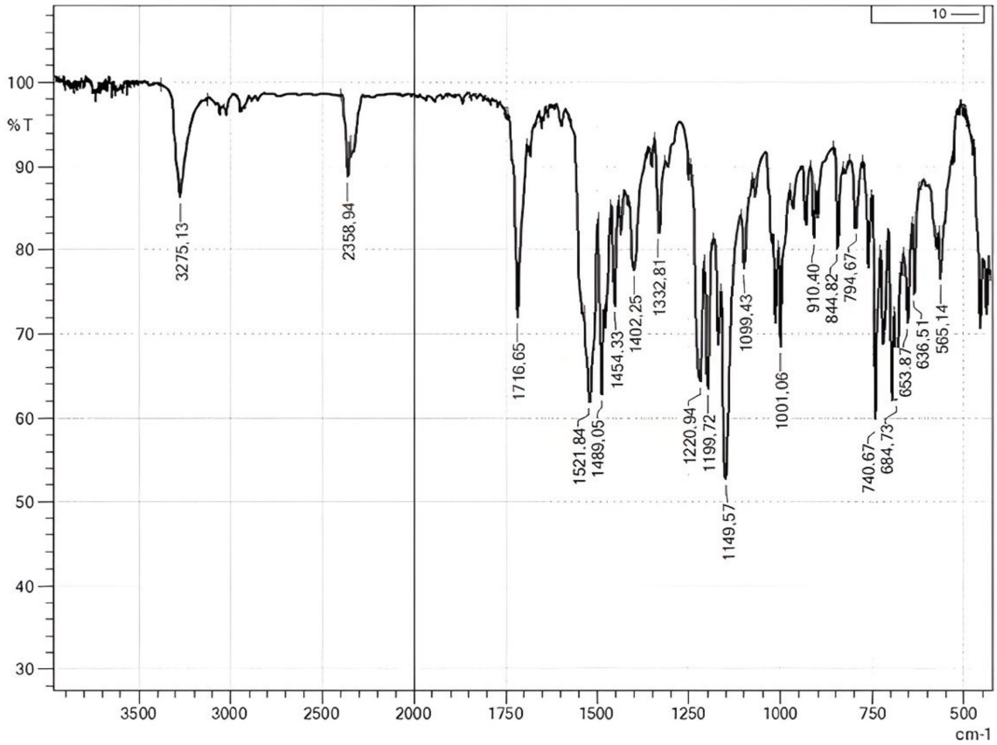

Figure S15. FTIR transmittance spectrum of N-phenethyl-N'-(phenoxycarbonyl) thiocarbamide **(10)**

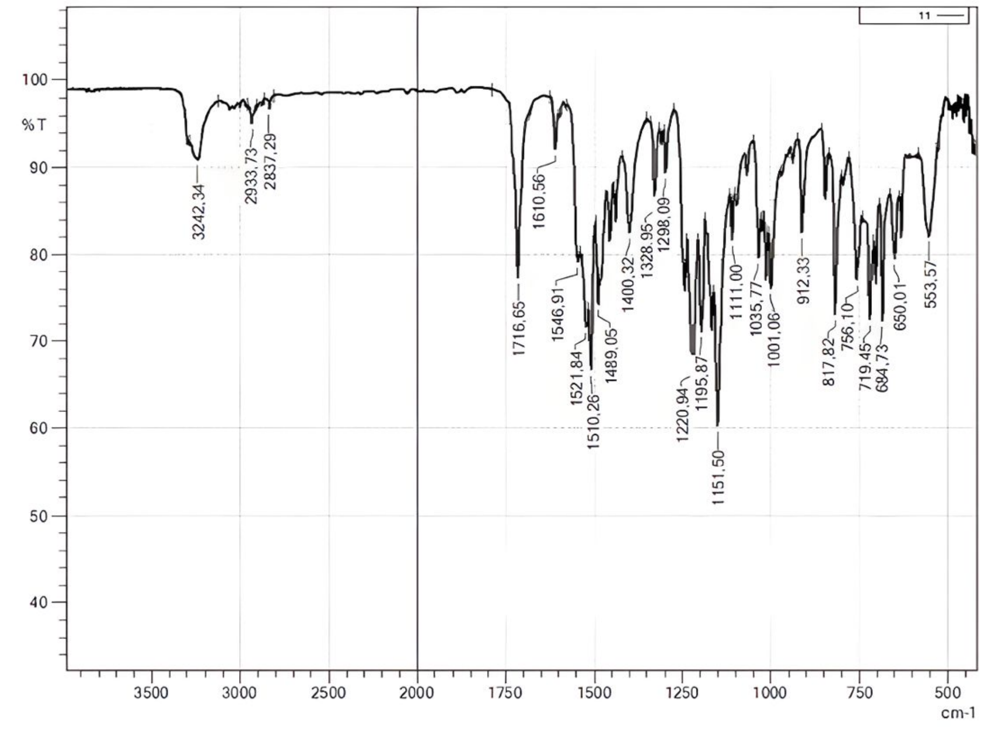

Figure S16. FTIR transmittance spectrum of N-(2-methoxyphenethyl)-N'-(phenoxycarbonyl) thiocarbamide **(11)**

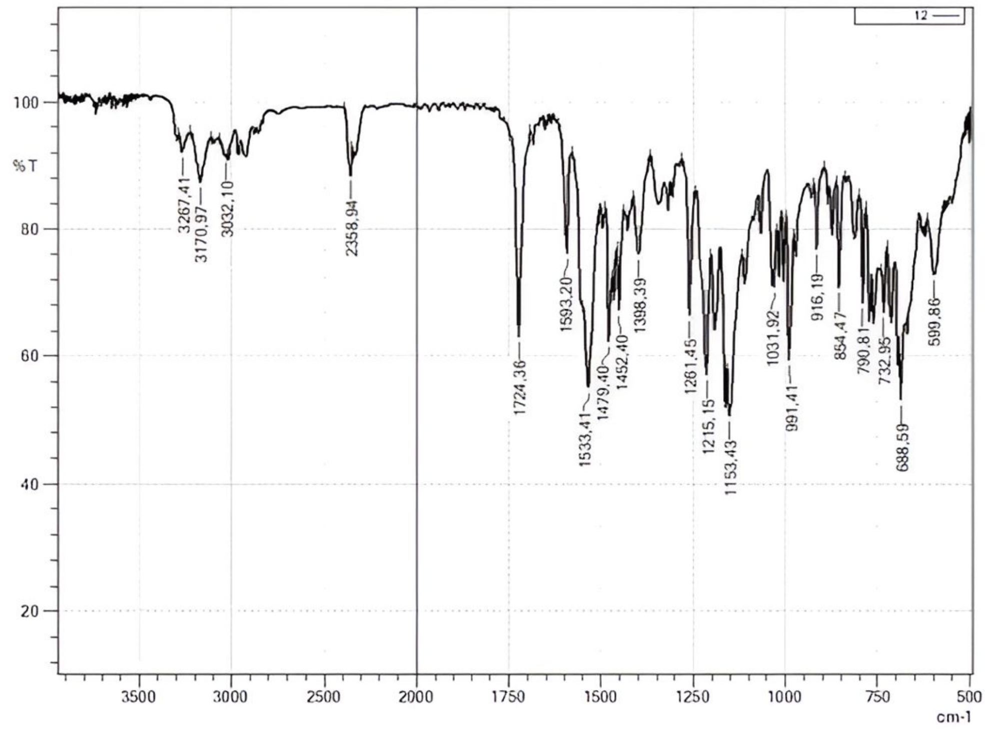

Figure S17. FTIR transmittance spectrum of N-(3-methoxyphenethyl)-N'-(phenoxycarbonyl) thiocarbamide **(12)**

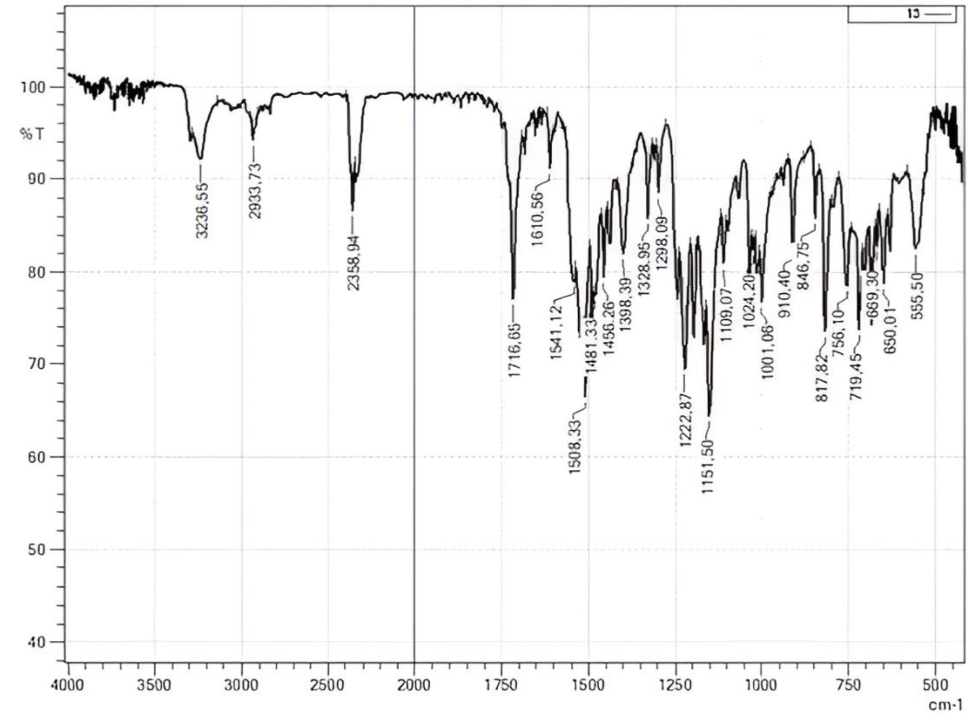

Figure S18. FTIR transmittance spectrum of N-(4-methoxyphenethyl)-N'-(phenoxycarbonyl) thiocarbamide **(13)**

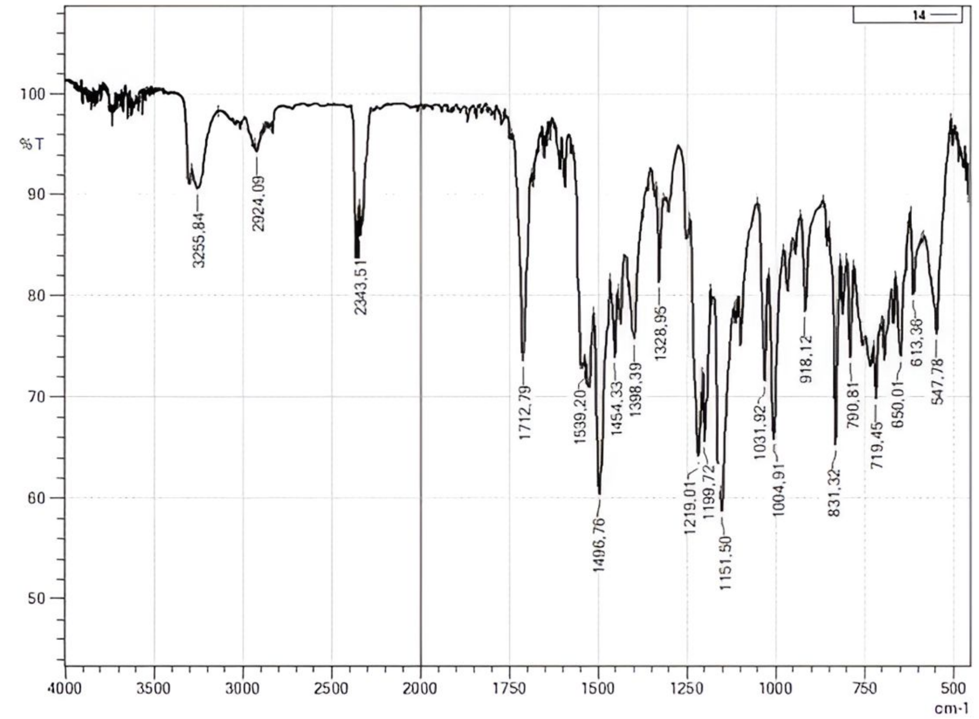

Figure S19. FTIR transmittance spectrum of N-(4-methylphenethyl)-N'-(phenoxycarbonyl) thiocarbamide **(14)**

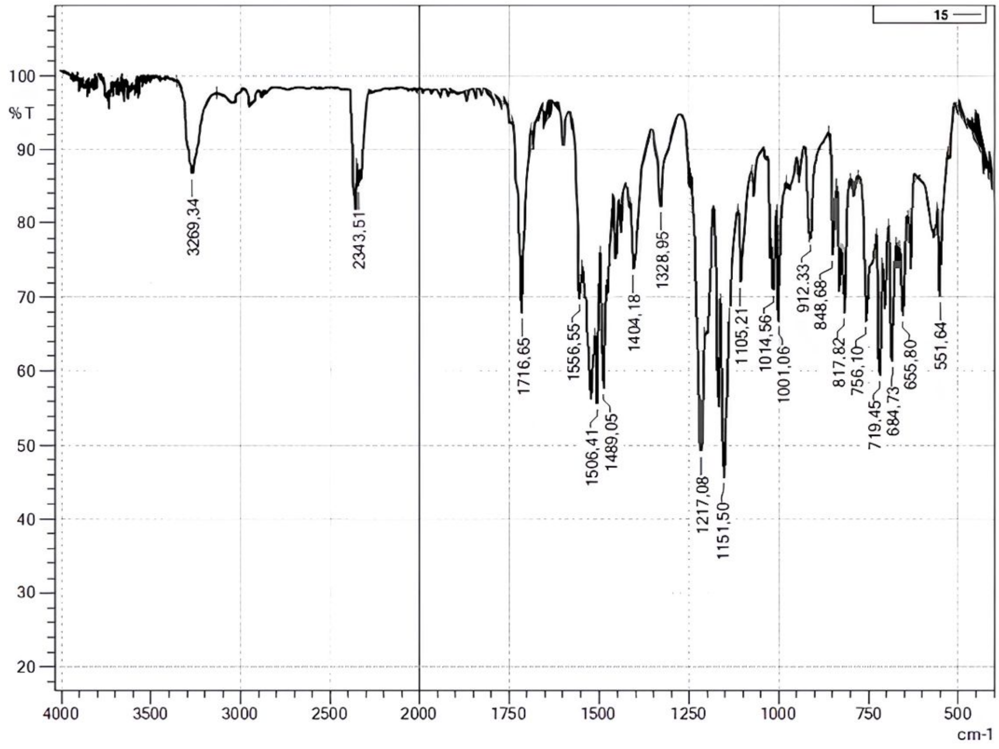

Figure S20. FTIR transmittance spectrum of N-(4-fluorophenethyl)-N'-(phenoxycarbonyl) thiocarbamide **(15)**

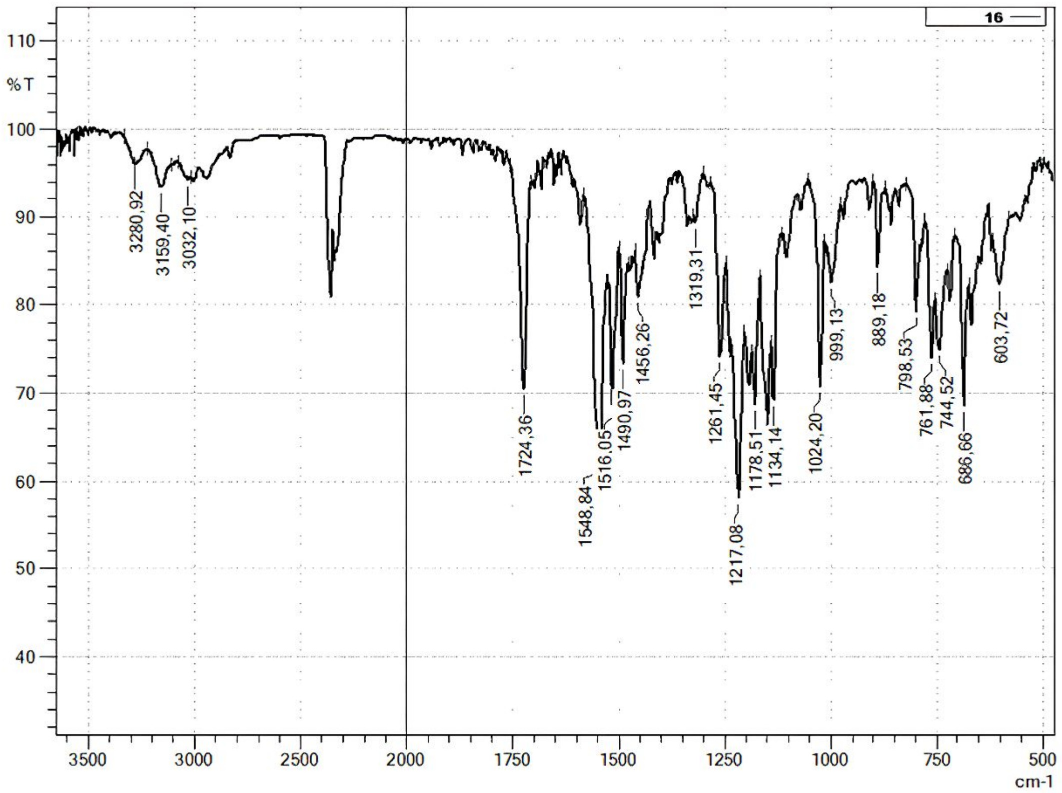

Figure S21. FTIR transmittance spectrum of N-(3,4-dimethoxyphenethyl)-N'-(phenoxycarbonyl) thiocarbamide **(16)**
